# Downregulation of castor zinc finger 1 predicts poor prognosis and facilitates hepatocellular carcinoma progression via MAPK/ERK signaling

**DOI:** 10.1186/s13046-018-0720-8

**Published:** 2018-03-05

**Authors:** Ji-Long Wang, Meng-yuan Yang, Shuai Xiao, Bo Sun, Yi-Ming Li, Lian-Yue Yang

**Affiliations:** 1 Liver Cancer Laboratory, Department of Surgery, Xiangya Hospital, Central South University, Xiangya Road 87, Changsha, Hunan 410008 China; 2 Department of Obstetrics and Gynecology, The Second Xiangya Hospital of Central South University, Changsha, Hunan China; 3 Department of Surgery, Xiangya Hospital, Central South University, Changsha, Hunan China

**Keywords:** Castor zinc finger 1, Hepatocellular carcinoma, Progression, MAPK/ERK, RAF1

## Abstract

**Background:**

Castor zinc finger 1 (CASZ1) plays critical roles in various biological processes and pathologic conditions, including cancer. However, the prognostic importance and biologic functions of CASZ1 in hepatocellular carcinoma (HCC) are still unclear.

**Methods:**

qRT-PCR, western blot and immunohistochemistry analyses were used to determine CASZ1 expression in HCC samples and cell lines. The clinical significance of CASZ1 was assessed in two independent study cohorts containing 232 patients with HCC. A series of in vitro and in vivo experiments were performed to explore the role and molecular mechanism of CASZ1 in HCC progression.

**Results:**

Here we report that CASZ1 expression was downregulated in HCC tissues and cell lines. Low CASZ1 expression was closely correlated with aggressive clinicopathological features, poor clinical outcomes and early recurrence of HCC patients. Moreover, overexpression of CASZ1 in HCCLM3 cells significantly inhibited cell proliferation, migration, invasion in vitro and tumor growth and metastasis in vivo, whereas silencing CASZ1 significantly enhanced the above abilities of PLC/PRF/5 cells. Further mechanism study indicated that these phenotypic changes were mediated by MAPK/ERK signaling pathway and involved altered expression of MMP2, MMP9 and cyclinD1. Finally, we proved that CASZ1 exerted its tumor-suppressive effect by directly interacting with RAF1 and reducing the protein stability of RAF1.

**Conclusions:**

Our study for the first time demonstrated that CASZ1 is a tumor suppressor in HCC, which may serve as a novel prognostic predictor and therapeutic target for HCC patients.

**Electronic supplementary material:**

The online version of this article (10.1186/s13046-018-0720-8) contains supplementary material, which is available to authorized users.

## Background

Hepatocellular carcinoma (HCC) is the second leading cause of cancer-related death worldwide, with more than 50% of HCC cancer cases and deaths occurred in China [1, 2]. Despite recent progress in HCC prevention, diagnosis and intervention, patients with HCC still have a very dismal long-term survival due to the high incidence of recurrence and metastasis [3, 4]. During the past decades, remarkable advances have been made to explore the pathogenesis of HCC [5–7]; but the underlying mechanisms responsible for HCC recurrence and metastasis are still largely unclear. Therefore, more reliable biomarkers for predicting relapse and understanding the mechanisms of HCC metastasis need to be developed urgently.

Castor zinc finger 1 (CASZ1) was initially characterized as a neural fate-determination gene [8], which plays critical roles in cell differentiation, as well as neural and cardiac developmental processes in Drosophila and Xenopus models [9–15]. In addition, CASZ1 promotes angiogenic sprouting and lumen morphogenesis through regulating endothelial cell contractility and adhesion [16, 17]. Recently, the importance of CASZ1 in tumorigenesis is becoming increasingly recognized. Aberrant fusion transcript of CASZ1 has been reported in colorectal cancer [18] and bladder cancer [19]. CASZ1 is downregulated and functions as a crucial tumor suppressor in neuroblastoma [20, 21]. In contrast, CASZ1 is highly expressed and responsible for cell migration and invasion in epithelial ovarian cancer [22], which indicated CASZ1 had different expression patterns and functions in various human cancers. Given the potential significance of CASZ1 in cancer pathobiology, its clinical relevance and potential role in human HCC deserves to be investigated.

In the present study, we found that CASZ1 was markedly downregulated in HCC tissues and cell lines, and low expression of CASZ1 was closely associated with aggressive clinicopathologic features, poor prognosis and early recurrence of HCC patients. Overexpression of CASZ1 suppressed, while silencing CASZ1 promoted HCC cell proliferation, migration and invasion both in vitro and in vivo. Furthermore, we demonstrated that CASZ1 inhibited MAPK/ERK signaling activity through direct downregulation of RAF1. Therefore, our results suggested that CASZ1 plays important suppressive roles in HCC progression and may serve as a potential prognostic predictor and therapeutic target for HCC.

## Methods

### HCC samples and follow-up

One hundred thirty-two HCC specimens in training cohort were randomly selected from Xiangya Hospital of Central South University between January 2007 and June 2010. Another 100 HCC samples in validation cohort were selected from the Affiliated Cancer Hospital of Xiangya School of Medicine between November 2008 and December 2011. The details of sample collection were shown in Additional file 1: Figure S1, while the baseline characteristics of these patients were described in Additional file 2: Table S1. All research protocols strictly complied with REMARK guidelines for reporting prognostic biomarkers in cancer [23]. Follow-up procedures were conducted as described in our previous study [24]. Frozen normal liver tissues (NLs), HCC tissues and adjacent non-tumor liver tissues (ANLTs) were used for qRT-PCR and western blot analysis.

### Cell lines and cell culture

MHCC97-L, MHCC97-H and HCCLM3 were kindly provided by the Liver Cancer Institute of Fudan University, Shanghai, China. PLC/PRF/5, Hep3B and HepG2 cells were purchased from the American Type Culture Collection (ATCC, Manassas, VA). L02, SMMC7721 and Huh7 cells were purchased from the Cell Bank of Typical Culture Preservation Committee of Chinese Academy of Science, Shanghai, China. Cells were maintained in Dulbecco’s Modified Eagle’s Medium (Biological Industries, Kibbutz Beit Haemek, Israel) supplemented with 10% fetal bovine serum (FBS, Biological Industries), 100 μg/mL streptomycin, and 100 U/mL penicillin (Hyclone, Logan, UT) at 37 °C in a humidified atmosphere with 5% CO_2_.

### RNA extraction and quantitative real-time PCR

Total RNA was extracted from tissues or cells with TRIzol reagent (Invitrogen,Carlsbad, CA). Reverse transcription were performed using an Advantage RT-for-PCR Kit (Takara, Dalian, China). qRT-PCR analysis was done using SYBR®Green Real time PCR Master Mix assay kit (Takara) in a 7300 Real-Time PCR system (Applied Biosystems Inc., Foster City, CA) with the following primers (5′ → 3′): CASZ1 forward, CCTCCCTGTCCTT CAACACT and reverse, GACGGCTGGTTTATCTGTGG; RAF1 forward, CCGAACAAGCAAAGAACAGTG and reverse, GACGCAGCATCAGTATTCCAAT. GAPDH was used as endogenous control.

### Protein extraction and western blot

Tissues or cells were lysed with RIPA buffer (Pierce, Rockford, IL) supplemented with protease inhibitors. Protein concentration was measured using a BCA protein assay (Thermo Scientific, Rockford, IL). Protein lysates, suspended in loading buffer, were separated on 10% SDS-polyacrylamide gels and transferred onto PVDF membranes (Millipore, Belford, MA). Then these membranes were blocked with 5% skim milk at room temperature for 1 h, and incubated with primary antibodies at 4 °C overnight. After washed, they were incubated with suitable HRP-conjugated secondary antibody at room temperature for 30 min and detected using an enhanced chemiluminescence (ECL) kit (Thermo Scientific). Antibodies for CASZ1, ERK and p-ERK were obtained from Abcam (Cambridge, MA), for RAF1, cyclinD1, E-cadherin, N-cadherin and Vimentin were purchased from Santa Cruz Biotechnology (Santa Cruz, CA), for MMP2 and MMP9 were purchased from Affinity Biosciences (Cincinnati, OH). β-actin antibody and corresponding secondary antibodies were purchased from Zhongshan Golden Bridge Biotechnology (ZSGB, Beijing, China).

### Immunohistochemical (IHC) analysis and scoring

The tissue sections were deparaffinized in xylene and rehydrated using a series of graded alcohols. Antigen retrieval was performed by microwave treatment in citrate buffer (pH 6.0) for 15 min. Endogenous peroxidase activity was inactivated using hydrogen peroxide (0.3%). After washed, the sections were blocked with 10% normal goat serum and incubated with primary antibody at 4 °C overnight. Signal was visualized using standard protocols with HRP conjugated secondary antibody and DAB (ZSGB) as the substrate. For negative controls, sections were incubated with normal goat serum rather than primary antibody. Finally, the sections were counterstained with hematoxylin and dehydrated in ethanol before mounting. The IHC score of target proteins was independently evaluated by two investigators according to the proportion and intensity of positive cells within five randomly selected fields per slide (magnification, × 400). The intensity was assessed by four grades: 0 for none; 1 for weak; 2 for moderate; 3 for strong. The percentage of positive cells was divided into five degrees: 0, no positive tumor cells; 1 for ≤5%; 2 for 6–25%; 3 for 26–75%; 4 for ≥76%. Immunoreactive score was calculated by multiplying the staining extent score with the intensity score. High expression was defined as a staining index score > 4, while low expression was defined as a staining index score ≤ 4 [25].

### Transfection and clone selection

The ectopic expression and knockdown lentivirus as well as control lentivirus for CASZ1 and RAF1 were all purchased from GenePharma (Suzhou, China). Transfection was performed according to the manufacturer’s instructions. Puromycin (2 μg/mL) was used to select stable clones. The sequences of shRNA are listed as follows (5′ → 3′): CASZ1-sh1: GCCGTCACTGAAGATGTAAAC; CASZ1-sh2: GCCAGTTCTACGGACAGAAGA; CASZ1-sh3: GCCCAGCAACGAATCAAATGG; RAF1-sh1: ATCAATTCAAGAGATTGATGT; RAF1-sh2: ACTTTTTCAAGAGAAAAGTTC; RAF1-sh3: CAATATTCAAGAGATATTGTT.

### MTT assay and colony formation assay

For MTT assay, 5 × 10^3^ cells were seeded in 96-well plates, incubated for 0–7 days, stained with MTT, and absorbance values were determined at 570 nm using a spectrophotometer. The relative cell number was normalized by the absorbance from the control cells. For colony formation assays, 500 cells were seeded per well in 6-well plates and cultured for 14 days. The colonies were fixed with 4% paraformaldehyde and stained with 1% crystal violet. Only colonies containing more than 50 cells were counted [26].

### Cell cycle analysis

5 × 10^5^ cells were seeded in 6-well plates and incubated for 24 h. Then cells were harvested and fixed with cold 70% ethanol at − 20 °C overnight. After washing, cells were stained in a solution containing PI (0.5 mg/mL) and RNase A (10 mg/mL). Then cells were filtered through a 70 μM cell strainer immediately prior to flow cytometry, which was carried out on a FACS caliber flow cytometer (BD Biosciences, San Jose, CA).

### Wound healing and transwell invasion assay

For wound healing assay, 5 × 10^5^ cells were seeded into 6-well plates and grown to confluence. Mitomycin C (10 μg/mL) was used to suppress cell proliferation before scratching [27]. Wounds were created by scraping the confluent cell monolayers with a 10 μL pipette tip. After extensively rinsed to remove cellular debris, cells were cultured in serum-free medium. The wound closure rate was monitored every 12 h and images were taken using an inverted microscope TE-2000S (Nikon, Tokyo, Japan). Transwell invasion assay was performed in a 24-well transwell plate with 8-μm polyethylene terephthalate membrane fiters (Corning Costar Corp, Corning, NY). 1 × 10^5^ cells in 200 μL of serum-free medium were added to the upper chambers, which contained matrigel-coated membranes (BD Biosciences). Each lower chamber was filed with 500 μL medium with 10% FBS. After 18 h or 24 h of incubation, cells that invaded to the bottom chamber were fixed with 4% paraformaldehyde and stained with 0.1% crystal violet. Invasive cells were counted in five randomly chosen fields (magnification, × 200) per well.

### Cancer 10-pathway reporter arrays

A Cignal Finder 10-Pathway Reporter Array (SABiosciences, Valencia, CA) was performed to explore the signaling pathways that were regulated by CASZ1 in HCC cells. The assay was conducted according to the manufacturer’s protocol. Relative firefly luciferase activity was calculated and normalized to the constitutively expressed Renilla luciferase. Experiments were done in triplicates.

### Co-immunoprecipitation (co-IP) assay and Cycloheximide (CHX) chase assay

For Co-IP, pre-cleared protein from whole cell lysates were incubated with antibody against CASZ1 or RAF1 at 4 °C overnight, which was conjugated to AminoLink Plus Resin (Pierce, Rockford, IL), The IP targets were disassociated from the immobilized antibodies on the AminoLink Plus Resin by the gentle elution buffer. Eluted proteins were resolved using 10% SDS-PAGE, followed by western blot with appropriate antibodies. Cycloheximide (CHX) chase assay was used to determine the half-life of RAF1. CASZ1-interfered HCC cells were treated with CHX (25 μg/mL) for the indicated times, and then were harvested and analyzed by western blot as described above. RAF1 protein degradation rates were quantified by densitometry using time point zero as 100%.

### Cell immunofluorescence staining

Indicated HCC cells (5 × 10^4^ cells) were seeded into 6-well plate with glass coverslips for 24 h. Then cells were successively fixed in 4% paraformaldehyde, permeabilized with 0.2% Triton X-100, blocked with 1% BSA and incubated with primary antibody at 4 °C overnight. After washed with PBS, cells were incubated with appropriate DyLight-conjugated secondary antibody and DAPI (Vector laboratories, Burlingame, CA). Finally, the slides were mounted and images were captured using an inverted fluorescence microscope DMI4000-B (Leica).

### HCC mouse models

Animal xenograft assays were conducted with 6-week-old male BALB/c nude mice (six mice per group). 5 × 10^6^ indicated cells were subcutaneously injected into the right dorsal flank of nude mice. Tumor sizes were measured at the indicated time points and calculated with the following formula: Tumor volume = L × W^2^ × 0.5 (L, length; W, width) [28]. After 4 weeks, the mice were sacrificed, and the tumors were obtained to weigh and undergo further experiments. Orthotopic tumor implantation was performed as described previously [29]. Tumor formation and metastatic progression was monitored and quantified using the Xenogen IVIS imaging system 100 (Caliper Lifescience, Hopkinton, MA). After 8 weeks, the mice were sacrificed, and the livers and lungs were harvested, imaged and processed for histopathological examination. All animal experiments were approved by the Institutional Animal Care and Use Committee of Central South University.

### Statistical analysis

Statistical analysis was performed using SPSS 18.0 software (SPSS Inc., Chicago, IL). The experimental data was presented as the mean ± SD and analyzed using Student’s *t* test or one-way ANOVA. The Chi-squared test was applied to examine the association between CASZ1 expression and clinical pathological parameters. Survival curves for patients were calculated using the Kaplan-Meier method and analyzed using the log-rank test. Prognostic factors were examined by univariate and multivariate analyses using the Cox proportional hazards model. Spearman’s rank analysis was performed to determine the correlation between different protein levels. All differences were deemed statistically significant at *P* < 0.05.

## Results

### CASZ1 is downregulated in human HCC tissues and cell lines

**Fig. 1:**
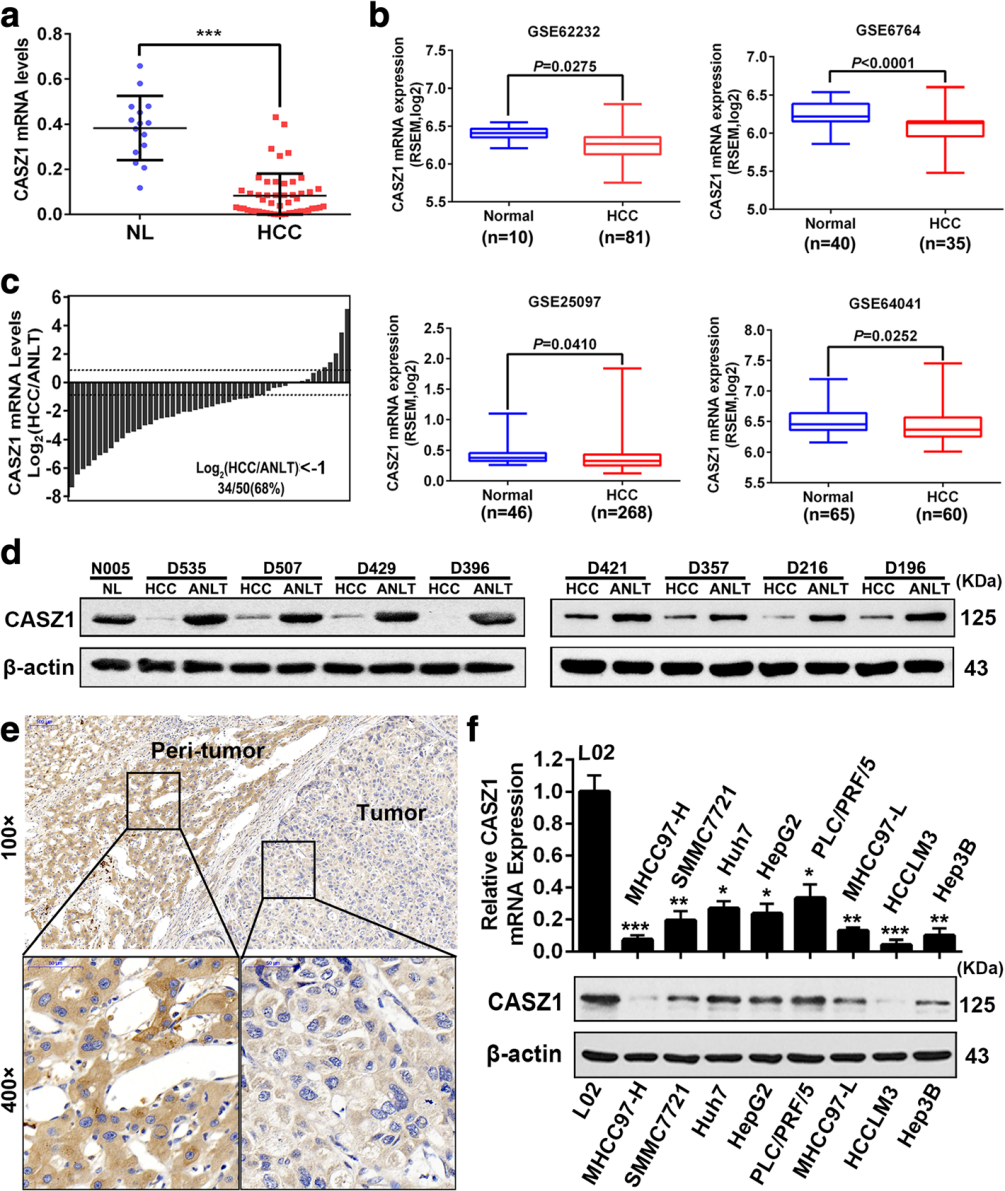
CASZ1 is downregulated in human HCC tissues and cell lines **a**. CASZ1 mRNA expression in 15 normal liver tissues (NLs) and 50 HCC tissues was analyzed by qRT-PCR. Data are shown as mean ± SD. ***P < 0.001. **b** CASZ1 expression was lower in HCC tissues than NLs according to the analysis of data from GEO (GSE62232, GSE6764, GSE25097, GSE64041, all *P* < 0.05). **c** Waterfall plot showing the downregulation of CASZ1 in 34 of 50 (68%) HCC samples compared to their matched adjacent non-tumor liver tissues (ANLTs). **d** The CASZ1 protein levels in HCCs, ANLTs and NLs were analyzed by Western blot. β-actin was used as a loading control. **e** Representative immunohistochemical images demonstrated CASZ1 protein was lowly expressed in HCC tumor tissues compared with their peri-tumor tissues. Magnification, × 100, × 400. **f** Expression of CASZ1 in HCC cell lines and L02, the normal liver cell line, were measured by qRT-PCR and western blot, respectively. *P < 0.05; **P < 0.01; ***P < 0.001 compared with L02

To determine the level of CASZ1 mRNA in HCC, qRT-PCR was performed in 15 normal liver tissues (NLs) and 50 pairs of HCC samples. Results showed that CASZ1 mRNA in HCC tissues was significantly lower than in the NLs (Fig. 1a), which was further verified by the analyses of mRNA sequencing datasets from Gene Expression Omnibus (Fig. 1b, GSE62232, GSE6764, GSE25097 and GSE64041, all *P* < 0.05). In addition, downregulation of CASZ1 (ANLT/HCC > 2) was detected in 34 of 50 (68%) HCCs compared to their matched adjacent non-tumor liver tissues (ANLTs) (Fig. 1c). Western blot analysis further confirmed CASZ1 expression was decreased in HCC tissues at protein level (Fig. 1d). Immunohistochemical analysis revealed that CASZ1 staining, mainly localized to the cytoplasm in the hepatic cells, was weaker in HCC tumor tissues than in their peri-tumor counterparts (Fig.1e). Moreover, we further detected the mRNA and protein levels of CASZ1 in eight HCC cell lines (MHCC97-H, SMMC7721, Huh7, HepG2, PLC/PRF/5, MHCC97-L, HCCLM3, Hep3B) and L02, an immortalized human liver cell line. Consistent with the above findings in HCC samples, our data showed that all examined HCC cell lines displayed lower mRNA and protein levels of CASZ1 than L02, especially for those (HCCLM3, HCC97-H, and Hep3B) with high invasion potential (Fig. 1f), which indicated a negative association between CASZ1 expression and HCC cell invasive ability. In summary, these results suggested that CASZ1 is downregulated in HCC, thus it may play an important role in HCC development.

### Low expression of CASZ1 is associated with aggressive clinicopathological characteristics and poor prognosis

**Fig. 2:**
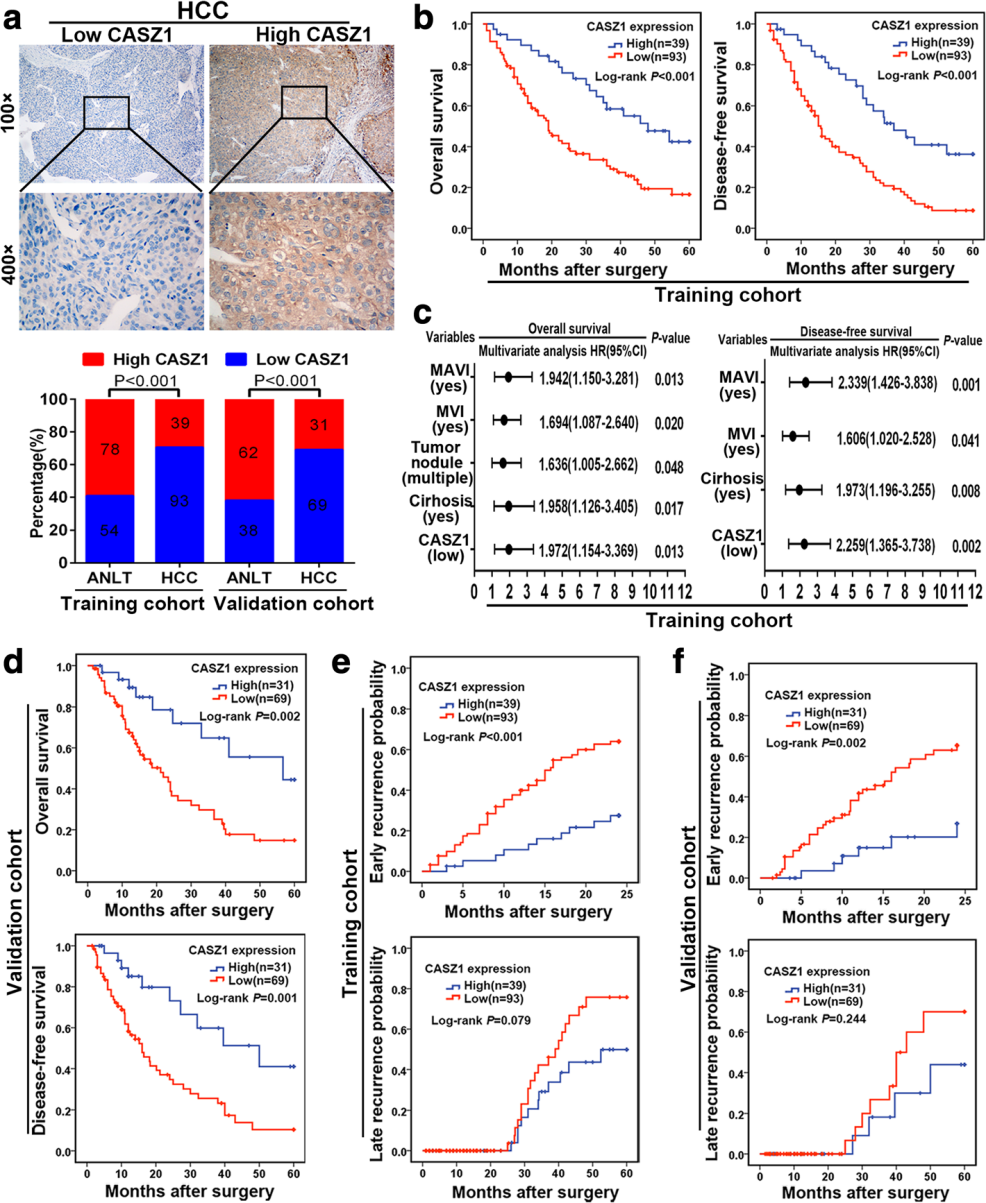
Low expression of CASZ1 is associated with aggressive clinicopathological characteristics and poor prognosis **a**. Representative images of low CASZ1 expression cases and high CASZ1 expression cases were shown (upper panel). Magnification, × 100, × 400. The percentages of low or high CASZ1 in paired HCC samples from the training and validation cohorts were compared (lower panel). **b** Kaplan-Meier analysis of OS and DFS based on CASZ1 expression in the training cohort. **c** Forest plots showing HR of OS and DFS for HCC patients in the indicated clinical subgroups of training cohort. **d** Kaplan-Meier analysis of OS and DFS based on CASZ1 expression in the validation cohort. **e** Kaplan-Meier analysis of early recurrence and late recurrence based on CASZ1 expression in the training cohort. **f** Kaplan-Meier analysis of early recurrence and late recurrence based on CASZ1 expression in the validation cohort

To explore the clinical significance of CASZ1 expression in HCC, we scored the IHC staining of HCC clinical samples from two independent cohorts: the training cohort (*n* = 132) and the validation cohort (*n* = 100), and defined CASZ1 expression as low or high according to the scores mentioned in Methods. In these two cohorts, we found that the percentage of low CASZ1 was significantly higher in HCC tissues than in ANLTs (Fig. 2a). On clinicopathological correlation, CASZ1 expression was significantly associated with tumor size (*P* = 0.021), tumor nodule number (*P* = 0.033), capsulation formation (*P* < 0.001), Edmondson-Steiner grade (*P* = 0.005), BCLC stage (*P* = 0.027) and TNM stage (*P* = 0.020) in the training cohort, however, there was no statistical correlation between CASZ1 expression and other clinicopathologic parameters, such as sex, gender, microvascular invasion (MVI), macrovascular invasion (MAVI) and so on (*P* > 0.05) (Additional file 2: Table S2). Importantly, these findings were further confirmed in the validation cohort (Additional file 2: Table S2). Kaplan-Meier survival analysis in the training cohort showed that HCC patients with low CASZ1 had shorter OS (median OS: 19.0 *v.s* 48.0 months; *P* < 0.001) and DFS (median DFS: 15.7 *v.s* 37.0 months; *P* < 0.001) than those with high CASZ1 (Fig. 2b). In addition, multivariate analysis proved low CASZ1 as an independent risk factor for both OS (HR = 1.972; 95% CI: 1.154–3.369; *P* = 0.013) and DFS (HR = 2.259; 95% CI: 1.365–3.738; *P* = 0.002) in HCC patients (Fig. 2c and Additional file 2: Table S3). Consistent with these results, in the validation cohort, we also found that CASZ1 expression inversely correlated with poor OS and DFS, and served as an independent prognostic marker in HCC patients (Fig. 2d and Additional file 2: Table S4). Of note, when tumor recurrence was classified as early recurrence and late recurrence using 2 year as the cutoff, we observed that the prognostic significance of CASZ1 was existed in the early recurrence group (*P* < 0.001), but not in the late recurrence group (*P* = 0.079) (Fig. 2e), which was consistent with the results from validation cohort (Fig. 2f). Thus, low CASZ1 expression may be a predictor for HCC early recurrence. Taken together, the above findings indicated that CASZ1 is a potential prognostic marker for HCC patients, which may involve in HCC aggressiveness and metastasis.

### CASZ1 inhibits HCC cell proliferation, migration and invasion in vitro

**Fig. 3:**
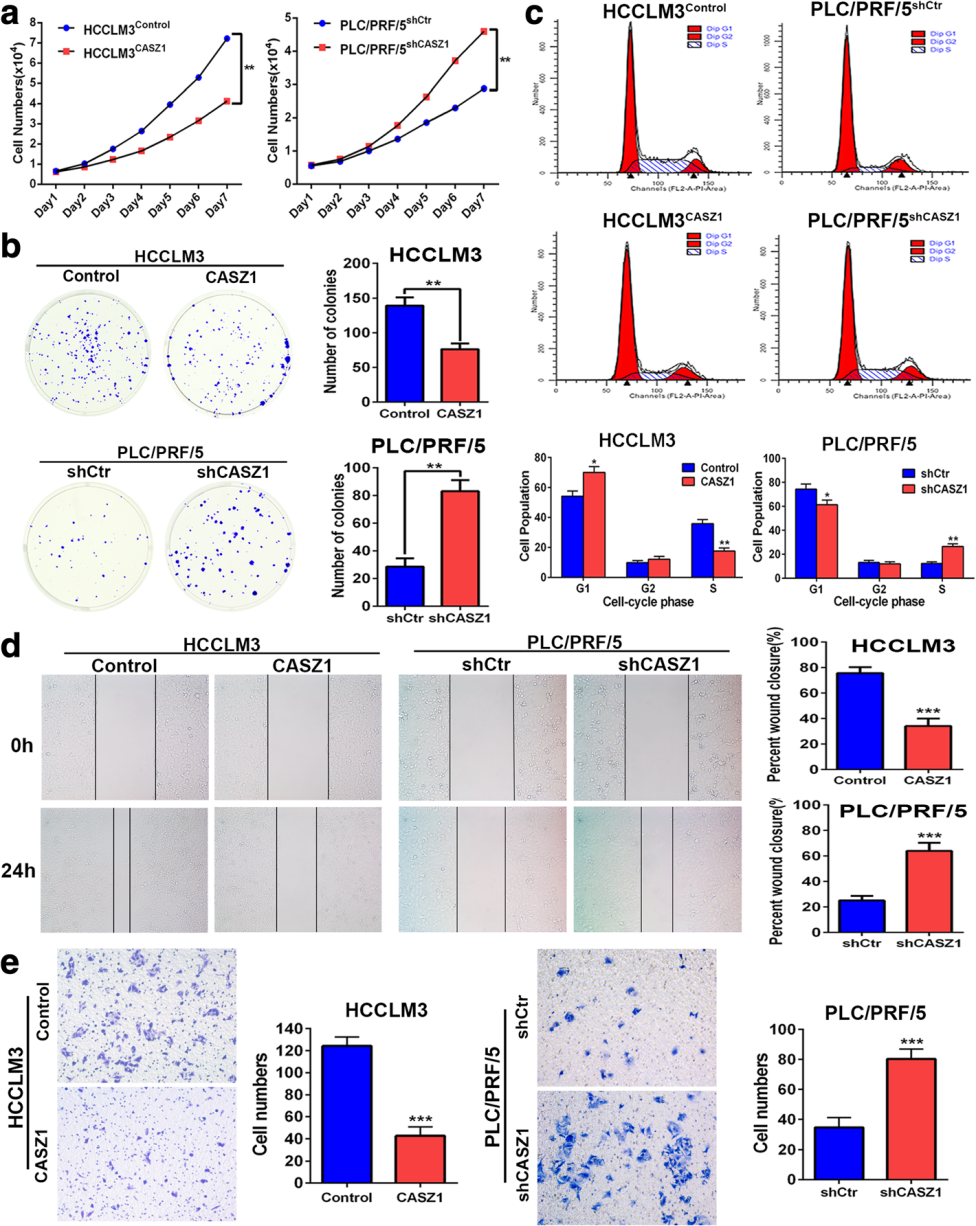
CASZ1 inhibits HCC cell proliferation, migration and invasion in vitro **a**. The effects of CASZ1 on HCC cell proliferation was measured by MTT assay at different time points. **b** Representative imgages and quantification of cell clones in HCCLM3^CASZ1^, PLC/PRF/5^shCASZ1^ and their control groups, as determined by colony formation assay. **c** The effect of CASZ1 on HCC cell cycle progression was analyzed by flow cytometry. **d** Wound healing assay was performed to detect the migratory capacity of HCCLM3^CASZ1^, PLC/PRF/5^shCASZ1^ and their control cells. **e** Transwell invasion assay was performed to evaluate the invasive potential of CASZ1-interfered HCC cells. Each bar represents the mean ± SD of three independent experiments. *P < 0.05; **P < 0.01; ***P < 0.001

To investigate the effects of CASZ1 on malignant phenotypes in HCC cells, we stably overexpressed CASZ1 in low CASZ1-expressing HCCLM3 cells, and knocked down it in high CASZ1-expressing PLC/PRF/5 cells using lentivirus transfection. The expression of CASZ1 in these resultant cells (HCCLM3^CASZ1^, HCCLM3^Control^, PLC/PRF/5^shCASZ1^ and PLC/PRF/5^shCtr^) were verified by qRT-PCR and western blot (Additional file 3: Figure S2A, B). Among the three shRNAs, we chose shRNA3, which achieved an 86% reduction in CASZ1 expression, for subsequent assays. Firstly, we analyzed the effects of CASZ1 on cell proliferation using MTT assay, which indicated that the cell proliferation rate was significantly decreased in HCCLM3^CASZ1^ group, but increased in PLC/PRF/5^shCASZ1^ group (Fig. 3a). Similarly, colony formation assay showed that overexpression of CASZ1 in HCCLM3 cells reduced the number of clones, whereas silencing CASZ1 in PLC/PRF/5 cells increased it (Fig. 3b). Next, we performed flow cytometry to investigate the effects of CASZ1 on cell cycle progression in HCC cells. As shown in Fig. 3c, overexpression of CASZ1 in HCCLM3 cells obviously increased the proportion of cells in G1 phase and decreased the proportion of cells in S phase, whereas knockdown of CASZ1 in PLC/PRF/5 cells resulted in the opposite trend, suggesting that CASZ1 may inhibit HCC cell proliferation by inducing cell cycle arrest at G1/S checkpoint. Furthermore, we investigated the potential role of CASZ1 in modulation of HCC cells to migrate and invade. In wound healing assay, we found that ectopic expression of CASZ1 decreased the rate of wound closure in HCCLM3 cells, whereas silencing CASZ1 in PLC/PRF/5 cells increased the rate of wound closure after scratch (Fig. 3d). Consistently, transwell invasion assays showed that HCCLM3^CASZ1^ group had less cells that passed through matrigel than HCCLM3^Control^ group, while PLC/PRF/5^shCASZ1^ group had more invasive cells than PLC/PRF/5^shCtr^ group (Fig. 3e). Collectively, these data strongly demonstrated that CASZ1 elicits a tumor-suppressive role in HCC progression by inhibiting cell proliferation, migration and invasion.

### CASZ1 represses HCC growth and metastasis in vivo

**Fig. 4:**
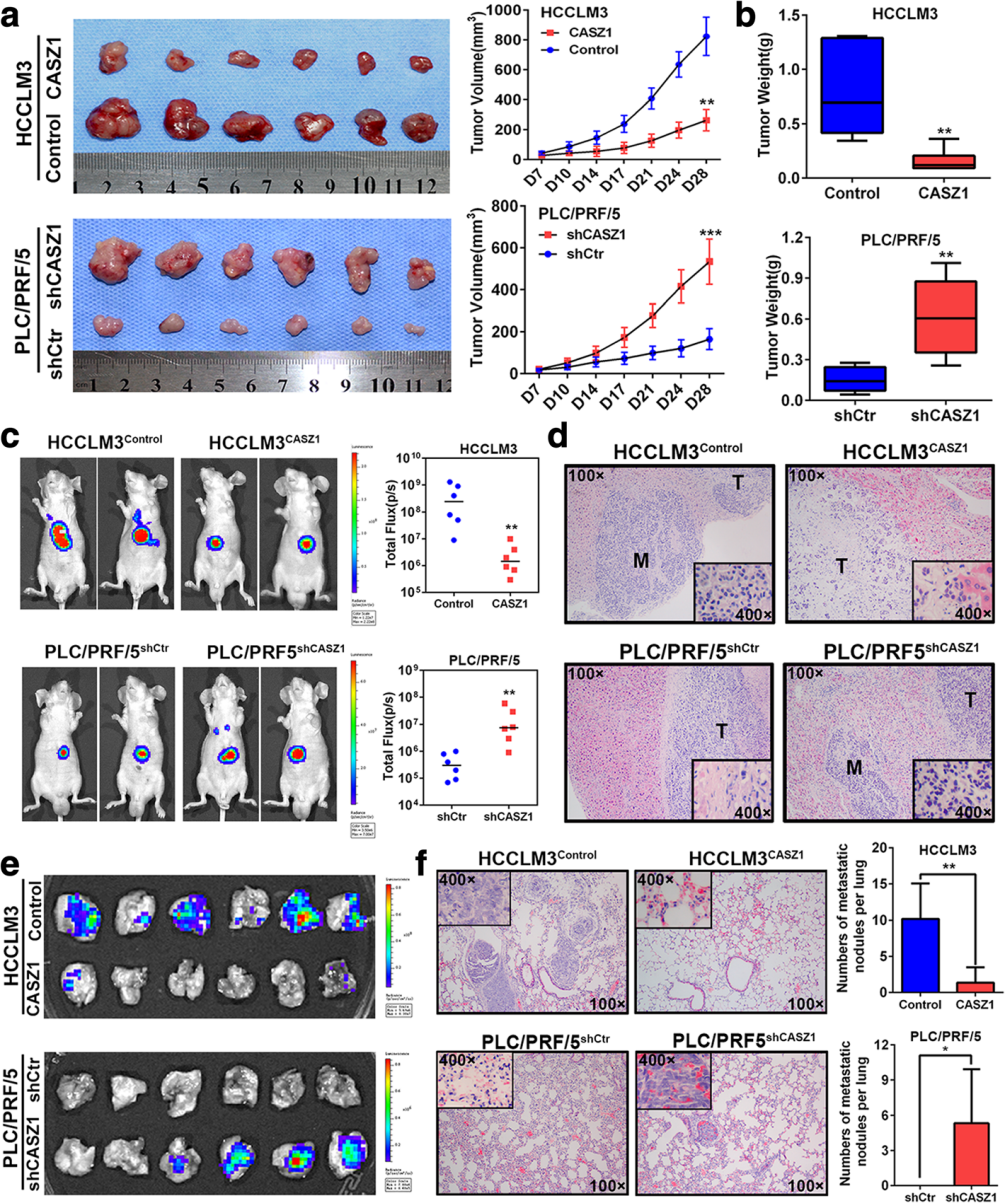
CASZ1 represses HCC growth and metastasis in vivo **a**. Effects of CASZ1 on HCC growth in subcutaneous xenograft model. The size of tumors was measured at indicated time points, and is shown as mean ± SD. **P < 0.01; ***P < 0.001. **b** Tumors were excised and weighted after mice were sacrificed. **P < 0.01. **c** Representative bioluminescent images of the orthotopic HCC model in HCCLM3^Control^, HCCLM3^CASZ1^, PLC/PRF/5^shCtr^ and PLC/PRF/5^shCASZ1^ groups (*n* = 6 for each group, left panel). The colored region represents the fluorescence signal of HCC cells in nude mice. Luciferase activity was quantified for experimental and control groups (right panel). **P < 0.01. **d** Representative H&E-stained sections of orthotopic primary liver tumors formed by HCCLM3^CASZ1^, PLC/PRF/5^shCASZ1^ and their control cells (original magnified × 100; inserted figures magnified × 400). **e** The presence of lung metastasis was examined in ex vivo bioluminescence imaging. **f** Representative H&E staining of pulmonary metastatic nodules in experimental and control groups (left panel: original magnified × 100; inserted figures magnified × 400). The number of lung metastatic nodules in each group is presented as mean ± SD (right panel). *P < 0.05; **P < 0.01

To verify the capacity of CASZ1 to inhibit HCC progression in vivo, we established subcutaneous xenograft tumor models and orthotopic xenograft tumor models using luciferase-labelled HCCLM3^CASZ1^, PLC/PRF/5^shCASZ1^ and their control cells. Consistent with the in vitro results, CASZ1 overexpression in HCCLM3 cells significantly inhibited tumor growth, as evidenced by tumor sizes and weights, whereas knockdown of CASZ1 in PLC/PRF/5 cells markedly increased tumor burden in vivo (Fig. 4a, b). Moreover, in liver orthotopic xenograft tumor models, IVIS imaging showed that tumors in HCCLM3^CASZ1^ group had weaker fluorescence signals than those in HCCLM3^control^ group, however, tumors from PLC/PRF/5^shCASZ1^ group exhibited stronger fluorescence signals than those from PLC/PRF/5^shCtr^ group (Fig. 4c), which indicated that CASZ1 could inhibit HCC growth and metastasis in vivo. H&E staining of liver tumors further confirmed the inhibitory role of CASZ1 in HCC intrahepatic metastasis, because tumors in HCCLM3^CASZ1^ group often had expansive tumor growth fronts with less invasive borders, whereas tumors from PLC/PRF/5^shCASZ1^ group exhibited invasive growth fronts with irregular tumor borders and tumor microsatellites (Fig. 4d). Moreover, ex-vivo bioluminescent imaging showed the incidence of lung metastasis was decreased in HCCLM3^CASZ1^ group, but increased in PLC/PRF/5^shCASZ1^ group (Fig. 4e**)**. Consistently, H&E staining confirmed that the number of pulmonary metastatic nodules was obviously decreased in HCCLM3^CASZ1^ group, but increased in PLC/PRF/5^shCASZ1^ group (*P* < 0.05, Fig. 4f). Together, these data provided strong evidence to support the tumor-suppressive function of CASZ1 in HCC growth and metastasis in vivo.

### CASZ1 inhibits HCC growth and metastasis via inactivating the MAPK/ERK signaling

**Fig. 5:**
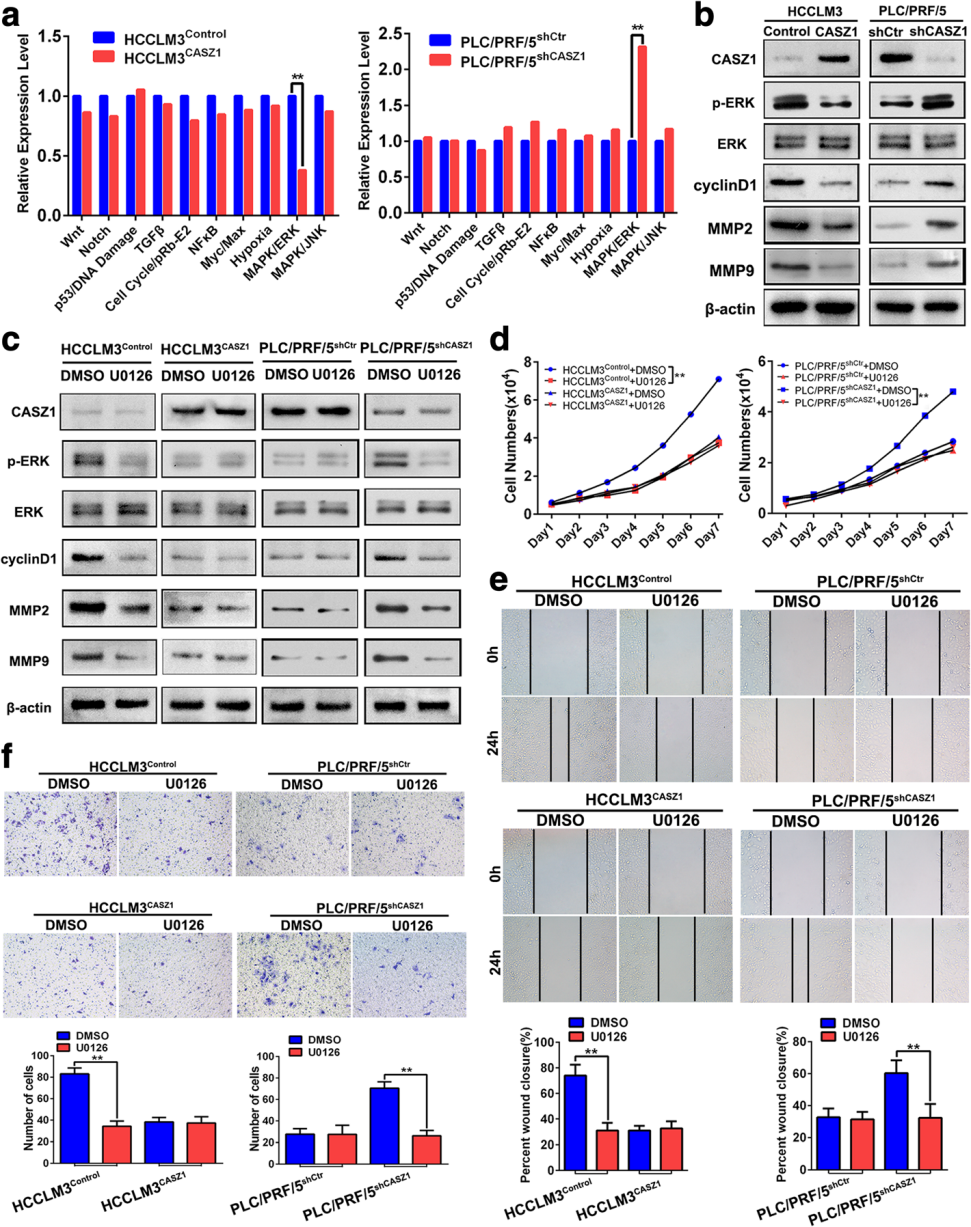
CASZ1 inhibits HCC growth and metastasis via inactivating the MAPK/ERK signaling **a**. 10-Pathway Reporter Array was performed to detect the signaling changes in CASZ1-interfered HCC cells. **P < 0.01. **b** The expression of critical members and downstream effectors of MAPK/ERK pathway was examined by western blot in HCCLM3^CASZ1^, PLC/PRF/5^shCASZ1^ and their control cells. **c** The levels of p-ERK, MMP2, MMP9 and cyclinD1 were determined by western blot in HCCLM3^CASZ1^, PLC/PRF/5^shCASZ1^ and their control cells treated with U0126 (10 μM) or DMSO control. **d**-**f**. HCCLM3^CASZ1^, PLC/PRF/5^shCASZ1^ and their control cells were treated with U0126 (10 μM) or DMSO control and then subjected to MTT (**d**), wound healing (**e**) and transwell invasion (**f**) assays

To probe the molecular mechanism underlying CASZ1-mediated inhibition of HCC growth and metastasis, we performed a Cignal Finder Cancer 10-Pathway Reporter Array in CASZ1 overexpression or knockdown HCC cells. Results showed that MAPK/ERK signaling pathway was the most significantly altered pathway when CASZ1 was interfered in HCC cells (Fig. 5a), indicating that CASZ1 may function via regulating the MAPK/ERK signaling. As expected, western blot analysis revealed that CASZ1 overexpression inhibited the phosphorylation of ERK in HCCLM3 cells, whereas CASZ1 knockdown resulted in an increase of p-ERK in PLC/PRF/5 cells, however, the total level of ERK kept unchanged (Fig. 5b). We further investigated the effect of CASZ1 on the expression of several genes related to cancer cell proliferation and invasion, such as MMP2, MMP9, cyclinD1 and epithelial-mesenchymal transition (EMT) genes, which were controlled by the MAPK/ERK pathway [30–32]. Data showed that the protein levels of MMP2, MMP9 and cyclinD1 were greatly decreased in CASZ1-ovexpressed HCCLM3 cells but increased in CASZ1-silenced PLC/PRF/5 cells (Fig. 5b). However, CASZ1 exhibited no significant effect on EMT gene expression and cell morphological change in HCC cells (Additional file 4: Figure S3A, B). IHC staining further demonstrated that the expression of p-ERK, MMP2, MMP9 and cyclinD1 were decreased in tumors induced by HCCLM3^CASZ1^ cells, whereas increased in tumors induced by PLC/PRF/5^shCASZ1^ cells (Additional file 4: Figure S3C). Moreover, we treated CASZ1-interfered HCC cells with U0126, a highly selective MEK inhibitor, and found that U0126 could obviously attenuated the promoting effect of low CASZ1 on the levels of p-ERK, MMP2, MMP9 and cyclinD1 in HCCLM3^Control^ and PLC/PRF/5^shCASZ1^ cells **(**Fig. 5c**)**. But due to the low level of phosphorylated ERK in the high CASZ1-expressing cells, further inhibition of ERK activity by U0126 didn’t affect the expression of p-ERK, MMP2, MMP9 and cyclinD1 in HCCLM3^CASZ1^ and PLC/PRF/5^shCtr^ cells **(**Fig. 5c**)**. Similarly, MTT, wound healing and transwell invasion assays also showed that U0126 markedly reduced the proliferative, migrative and invasive abilities of HCCLM3^Control^ and PLC/PRF/5^shCASZ1^cells, but produced no significant effects in high CASZ1-expressing cells (Fig. 5d-f**)**. Together, these data indicated that CASZ1 could negatively regulate MAPK/ERK signaling and its downstream effectors to inhibit HCC progression.

### CASZ1 decreases RAF1 expression by reducing the protein stability of RAF1

**Fig. 6:**
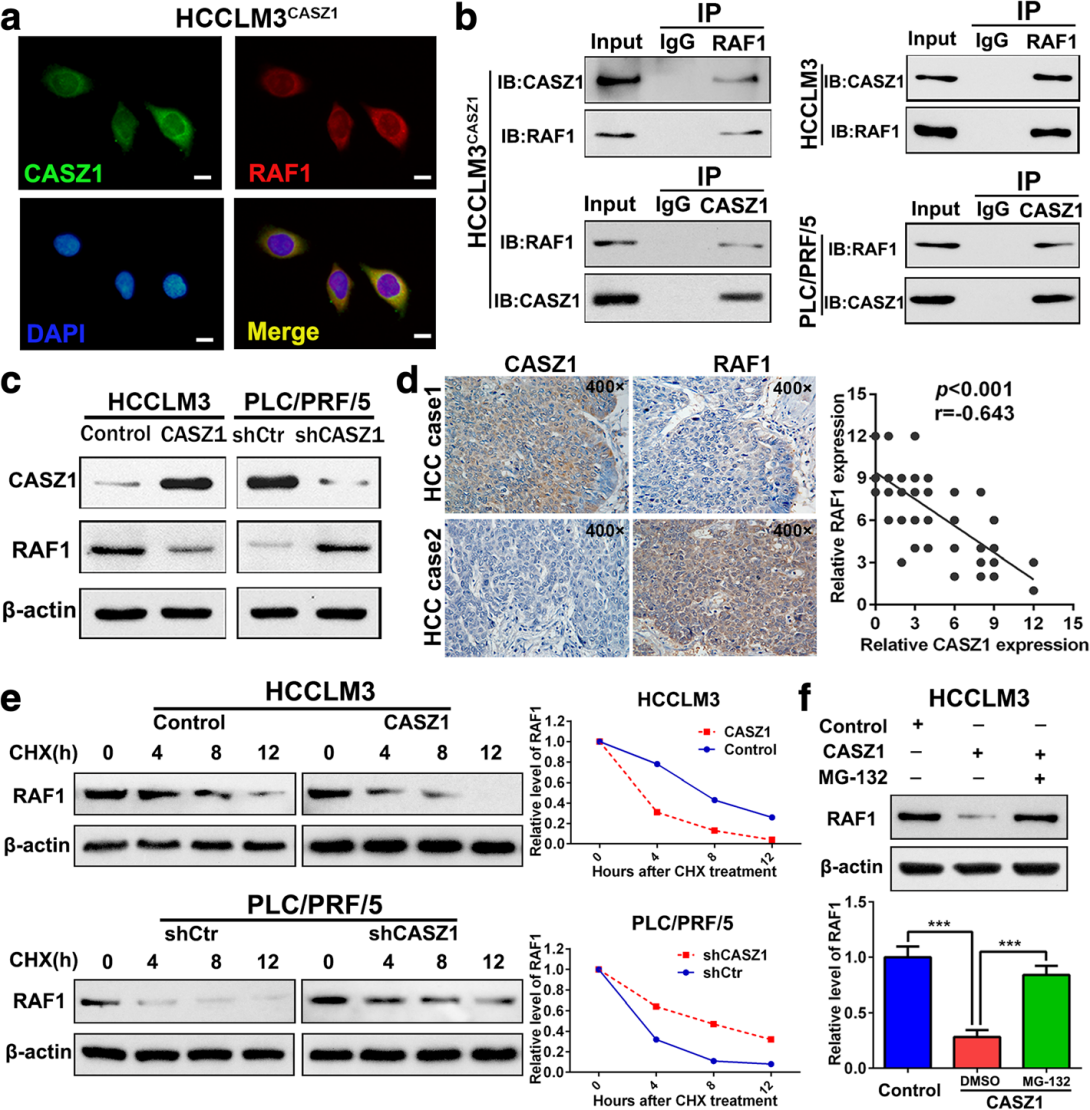
CASZ1 decreases RAF1 expression by reducing the protein stability of RAF1 **a**. HCCLM3 cells transfected with CASZ1 were fixed for the double immunofluorescence staining, and colocalization of CASZ1 with RAF1 were visualized as yellow fluorescence in merge panel. Scale bar, 25 μm. **b** Co-immunoprecipitation assay was performed to analyze the direct binding between CASZ1 and RAF1 in HCCLM3^CASZ1^, HCCLM3 and PLC/PRF/5 cells. **c** The expression of RAF1 was determined in CASZ1-interfered HCC cells by western blot. **d** Correlation between CASZ1 and RAF1 was measured in 50 HCC samples. Representative IHC staining of CASZ1 and RAF1 (left panel, magnification, × 400) in serial sections are shown, indicating a negative correlation between the protein level of CASZ1 and RAF1 in the clinical samples (*r* = − 0.643, *P* < 0.001, right panel). **e** The half-life of RAF1 protein in HCC cells was analyzed following treatment with cycloheximide (CHX, 25 μg/ml) for the indicated time points. The half-life of RAF1 protein was decreased in HCCLM3 cells with CASZ1 overexpression, but increased in PLC/PRF/5 cells with CASZ1 knockdown. **f** MG-132 (20 μM) was used to inhibit the proteasomal degradation in HCCLM3 cells. MG-132 treatment reversed the downregulation of RAF1 protein induced by CASZ1 overexpression. *** *P* < 0.001

To explore the molecular mechanism by which CASZ1 exerted its tumor-suppressive function in HCC cells, we reviewed literatures and searched BioGrid 3.4 database (Additional file 5: Figure S4A). We found that CASZ1 might interact with RAF1, a highly-conserved serine/threonine kinase of the MAPK pathway that involved in cell proliferation, transformation, survival and metastasis of various cancers [33–35]. As expected, immunofluorescence staining results showed that CASZ1 co-localized with RAF1 in the cytoplasm of CASZ1-transfected HCCLM3 cells (Fig. 6a), while co-IP results revealed CASZ1 and RAF1 could interact with each other in HCC cells (Fig. 6b). Next, we investigated whether the interaction between CASZ1 and RAF1 could affect RAF1 expression in HCC cells. Interestingly, we found that ectopic expression of CASZ1 in HCCLM3 cells decreased the protein level of RAF1, whereas silencing CASZ1 in PLC/PRF/5 cells increased RAF1 protein expression (Fig. 6c), however, manipulating CASZ1 expression had little effect on RAF1 mRNA expression (Additional file 5: Figure S4B). The above results indicated that CASZ1 downregulates RAF1 at the protein (but not mRNA) level. Moreover, IHC staining in 50 HCC samples randomly selected from the training and validation cohorts showed that RAF1 protein was highly expressed in HCC tissues and negatively correlated with CASZ1 expression (*P* < 0.001, *r* = − 0.643, Fig. 6d). Subsequently, we investigated whether CASZ1 decreased RAF1 level in HCC cells by regulating its protein stability. As shown in Fig. 6e, overexpression of CASZ1 could dramatically decreased the half-life of RAF1 protein after added translation inhibitor cycloheximide (CHX). Conversely, silence of CASZ1 remarkably prolong the half-life of RAF1 protein (Fig. 6e). Furthermore, MG132, the proteasome inhibitor, rescued the decrease of RAF1 induced by CASZ1 overexpression in HCCLM3 cells (Fig. 6f). These results indicated that CASZ1 decreases RAF1 expression in HCC cells by reducing the protein stability of RAF1.

### RAF1 is critical for CASZ1-mediated inhibition of MAPK/ERK signaling and HCC progression

**Fig. 7:**
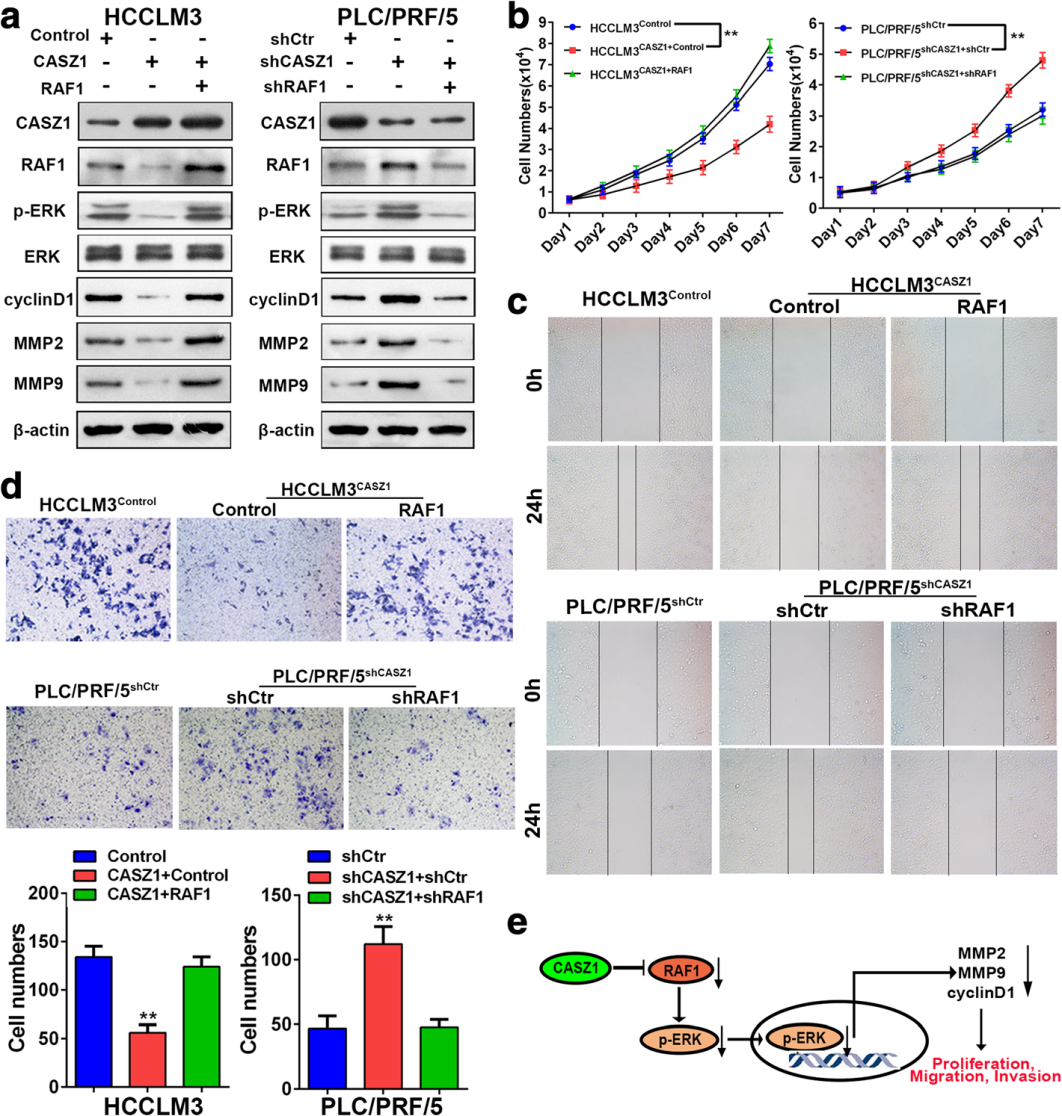
RAF1 is critical for CASZ1-mediated inhibition of MAPK/ERK signaling and HCC progression **a**. Western blot analysis of CASZ1, RAF1, p-ERK, MMP2, MMP9 and cyclinD1 expression in CASZ1-interfered HCC cells with RAF1 knockdown or ectopic expression. **b**-**d**. MTT (**b**), wound healing (**c**) and transwell invasion (**d**) assays were performed to test the influence of RAF1 on the proliferation, migration and invasion abilities of CASZ1-interfered HCC cells with RAF1 knockdown or ectopic expression. **e** Schematic depiction of the mechanism underlying CASZ1-mediated inhibition of HCC cell proliferation, migration and invasion based on our study

To test whether RAF1 is indispensable for CASZ1 to inactivate the MAPK/ERK signaling and inhibit HCC progression, we transfected the RAF1 ectopic expression plasmid into HCCLM3^CASZ1^ cells, and RAF1-shRNA into PLC/PRF5^shCASZ1^ cells. The ectopic expression and silence efficacy of RAF1 was varified by qRT-PCR and western blot, respectively (Additional file 6: Figure S5A, B). We found that the suppressive effect of CASZ1 on the levels of p-ERK, MMP2, MMP9 and cyclinD1 was significantly abrogated by RAF1 overexpression in HCCLM3^CASZ1^ cells, while the promotion effect of CASZ1 silence on these proteins expression was greatly inhibited by RAF1 knockdown in PLC/PRF/5^shCASZ1^ cells, however, altering RAF1 expression didn’t affect CASZ1 levels in HCC cells (Fig. 7a). These data indicated that RAF1 is necessary for the inactivation of MAPK/ERK signaling by CASZ1. Furthermore, the RAF1-dependency on CASZ1-mediated inhibition of HCC progression was also assessed by gain-and-loss function assays, which showed that overexpression of RAF1 in HCCLM3^CASZ1^ cells restored their proliferation and metastatic capacity, whereas knockdown of RAF1 in PLC/PRF/5^shCASZ1^ cells eliminated the promoting effect of CASZ1 silence on cell proliferation, migration and invasion (Fig. 7b-d and Additional file 6: Figure S5C). Together, these results proved that CASZ1 inhibits HCC growth and metastasis by regulating MAPK/ERK signaling via RAF1 in HCC cells (Fig. 7e).

## Discussion

Human CASZ1 gene is mapped on chromosome 1p36 and has a central role in diverse biological processes, such as neurogenesis, cardiac development and vascular morphogenesis [36]. Previous studies have reported the controversial roles of CASZ1 in several cancers [20, 22], which may be related to tumor and tissue specificity. However, to date, the relationship between CASZ1 and HCC progression is still unclear. In the present study, we proved that CASZ1 expression was obviously decreased in HCC tissues and cell lines. Overexpression of CASZ1 markedly inhibited HCC cell proliferation, migration and invasion in vitro, whereas knockdown of CASZ1 exerted the opposing effects. Interestingly, cell cycle analysis by flow cytometry showed that CASZ1 could induce cell cycle arrested at G1/S checkpoint. Furthermore, the in vivo animal experiments demonstrated that CASZ1 overexpression not only inhibits tumor growth, but also suppresses HCC metastasis, as determined by IVIS detection and H&E staining. Previous studies indicated that CASZ1 is a metastatic promoter in ovarian cancer [22], however, Liu et al. proved CASZ1 inhibits cell proliferation and migration in neuroblastoma [20, 21]. In the present study, our findings were in accordance with Liu’s report, and strongly demonstrated that CASZ1 is a tumor suppressor in HCC, which may be a potential therapeutic target for HCC.

In this study, we further explored the clinical significance of CASZ1 in two independent study cohorts consisting of 232 HCC subjects. We found that low CASZ1 significantly correlated with aggressive clinicopathologic features, such as larger tumor size, multiple tumor nodules, lack of capsule, higher Edmondson-Steiner grade, advanced BCLC stage and TNM stage, which further proved the suppressive role of CASZ1 in HCC progression. Moreover, survival analysis revealed that CASZ1 is an independent predictor for HCC prognosis, and low CASZ1 always indicated a shorter OS and DFS for HCC patients. Notably, we also observed that HCC patients in the low CASZ1 group had a significantly higher early recurrence rate than those from the high CASZ1 group, which suggested a potential predictive role of CASZ1 in HCC early recurrence. It’s well known that early recurrence is an important adverse prognostic factor, which mainly results from dissemination of metastatic HCC cells [37, 38], thus detecting CASZ1 expression may help to predict HCC progression, and direct personalize therapy for HCC patients in the future. To the best of our knowledge, this is the first report detailing CASZ1 as a prognostic predictor in HCC.

We then explored the molecular mechanism of CASZ1 to inhibit HCC progression. Although CASZ1 was reported to modulate EGFL7/RhoA pathway and pRb activity in different cell types [16, 39], however, given the context-dependent function of CASZ1 in various cancers, we speculated that CASZ1 may have a different mechanism to counteract HCC growth and metastasis. Indeed, the results of 10-Pathway Reporter Array in CASZ1-interfered HCC cells showed that CASZ1 could significantly inhibited the activity of MAPK/ERK signaling, which was further confirmed by western blot and IHC assays. Our data also revealed that CASZ1 significantly reduced the levels of MMP2, MMP9 and cyclinD1 in HCCLM3^CASZ1^ cells, but increased their expression in PLC/PRF/5^shCASZ1^ cells. As the downstream effectors of p-ERK, MMP2 and MMP9 are important for tumor invasion and metastasis by degrading basement membrane components [40], while cyclinD1 acts on G1-S progression of the cell cycle [41], thus they may decipher the function of CASZ1 in HCC proliferation and metastasis. Interestingly, although CASZ1 was reported to promote cell migration and invasion via EMT in ovarian cancer [22], however, in this study, we found that altering CASZ1 expression in HCC cells had no significant effects on the EMT gene expression and cell morphologic changes, indicating the role of CASZ1 in HCC progression was EMT-independent. To further examine whether the tumor-suppressive effects of CASZ1 were dependent on MAPK/ERK pathway, we treated CASZ1-interfered HCC cells with a MEK inhibitor U0126. Data showed that U0126 could effectively decrease the expression of p-ERK, MMP2, MMP9 and cyclinD1 in low CASZ1-expressing HCC cells. Moreover, it also abolished low CASZ1-induced proliferative, migratory and invasive ability in HCCLM3^Control^ and PLC/PRF/5^shCASZ1^ cells. Taken together, the above findings indicated that CASZ1 inhibits HCC progression via inactivating the MAPK/ERK signaling.

RAF1 is an important kinase that transduces signals from the cell membrane to nucleus and activates MAPK/ERK pathway [42]. Accumulating evidences indicate that RAF1 is highly expressed in various malignant tumors, including HCC [35, 43, 44]. Moreover, Chen et al. reported that increased RAF1 in HCC is significantly correlated with poor prognosis [45]. In this study, we demonstrated that RAF1 was negatively regulated by CASZ1 in HCC cells, and there is an inverse correlation between CASZ1 and RAF1 protein levels in HCC clinical specimens. Importantly, we also proved that CASZ1 could interact with RAF1 and attenuate RAF1 expression by regulating its protein stability in HCC cells, however, the exact mechanism for CASZ1-mediated protein degradation of RAF1 requires further investigation in the future. Moreover, we found that CASZ1-induced RAF1 downregulation contributed to the inhibitory effects of CASZ1 on the MAPK/ERK signaling and HCC progression. Because ectopic expression of RAF1 effectively impeded the ability of CASZ1 to inactivate MAPK/ERK signaling and inhibit cell proliferation, migration and invasion in HCCLM3 cells, whereas silencing RAF1 offset these promoting effects of CASZ1 knockdown in PLC/PRF/5 cells. Thus, our study provided evidence to support that CASZ1 exerts its inhibitory function in HCC progression through regulating MAPK/ERK pathway via RAF1.

## Conclusions

In summary, we proved that CASZ1 expression is downregulated in HCC tissues, which significantly correlated with aggressive clinicopathological characteristics, poor prognosis and early recurrence of HCC patients. Furthermore, CASZ1 inhibits HCC cell proliferation, migration and invasion through regulating MAPK/ERK signaling via RAF1. Thus these findings together defined CASZ1 as a tumor suppressor in HCC, which may be a novel prognostic biomarker and therapeutic target for HCC patients.

## Additional files

Additional file 1: **Figure S1.** Flow chart showing the details for selecting HCC samples in this study. (TIFF 1194 kb)

Additional file 2: **Table S1.** Clinicopathologcal characteristics of HCC patients in training cohort and validation cohort. **Table S2** Correlation between CASZ1 expression and clinicopathologic characteristics of HCC patients in training cohort and validation cohort. **Table S3** Univariate and multivariate analyses of risk factors associated with overall survival and disease-free survival of HCC patients in training cohort. **Table S4** Univariate and multivariate analyses of risk factors associated with overall survival and disease-free survival of HCC patients in validation cohort. (DOC 282 kb)

Additional file 3: **Figure S2.** The efficacy of CASZ1 ectopic expression or silence was determined in HCC cells. **A**-**B**. qRT-PCR (**A**) and western blot (**B**) confirmed CASZ1 mRNA and protein levels in HCCLM3^CASZ1^, PLC/PRF/5^shCASZ1^ and their respective control cells. (TIFF 8263 kb)

Additional file 4: **Figure S3.** CASZ1 inhibits HCC progression by inactivating the MAPK/ERK pathway. **A** EMT genes including E-cadherin, N-cadherin and vimentin were detected by western blot in HCCLM3^CASZ1^, PLC/PRF/5^shCASZ1^ and their control cells. **B** Cell morphological changes in HCCLM3^CASZ1^, PLC/PRF/5^shCASZ1^ and their control cells was examined by phase-contrast photomicrographs. **C** IHC staining showed that the expression of p-ERK, cyclinD1, MMP2 and MMP9 was reduced in the CASZ1-overexpressed HCCLM3 xenograft tumors, but increased in the CASZ1-silenced PLC/PRF/5 xenograft tumors (magnification, × 400). (TIFF 11458 kb)

Additional file 5: **Figure S4.** CASZ1 may interact with RAF1 in HCC cells. **A** Potential CASZ1-interacting partners were analyzed using BioGRID3.4 (https://thebiogrid.org). **B** The expression of RAF1 mRNA was determined in CASZ1-interfered HCC cells by qRT-PCR. (TIFF 6522 kb)

Additional file 6: **Figure S5.** The efficacy of RAF1 ectopic expression or silence is determined in CASZ1-interfered HCC cells. **A**-**B**. qRT-PCR (**A**) and western blot (**B**) confirmed RAF1 mRNA and protein levels in HCCLM3^CASZ1^ cells with RAF1 overexpression or PLC/PRF/5^shCASZ1^ cells with RAF1 knockdown. C. The wound closure rate of CASZ1-interfered HCC cells with RAF1 ectopic expression or knockdown. * *P* < 0.05, ** *P* < 0.01. (TIFF 2192 kb)

## Abbreviations

AFP: alpha-fetoprotein
ANLT: adjacent non-tumor liver tissue
BCLC: Barcelona Clinic Liver Cancer
CASZ1: castor zinc finger 1
DFS: disease-free survival
ERK: extracellular signal-regulated kinase
GAPDH: glyceraldehyde-3-phosphate dehydrogenase
H&E: hematoxylin and eosin
HCC: hepatocellular carcinoma
IHC: immunohistochemistry
MAPK: mitogen-activated protein kinase
MAVI: macrovascular invasion
MMP: matrix metalloproteinase
MVI: microvascular invasion
OS: overall survival
RAF1: raf-1 proto-oncogene, serine/threonine kinase
shRNA: short-hairpin RNA
TNM: tumor-node-metastasis
