# Supplementary figures and images for: Downregulation of castor zinc finger 1 predicts poor prognosis and facilitates hepatocellular carcinoma progression via MAPK/ERK signaling
Source: J Exp Clin Cancer Res. 2018 Mar 5;37:45. doi: 10.1186/s13046-018-0720-8 (PMC5836448; doi:10.1186/s13046-018-0720-8)

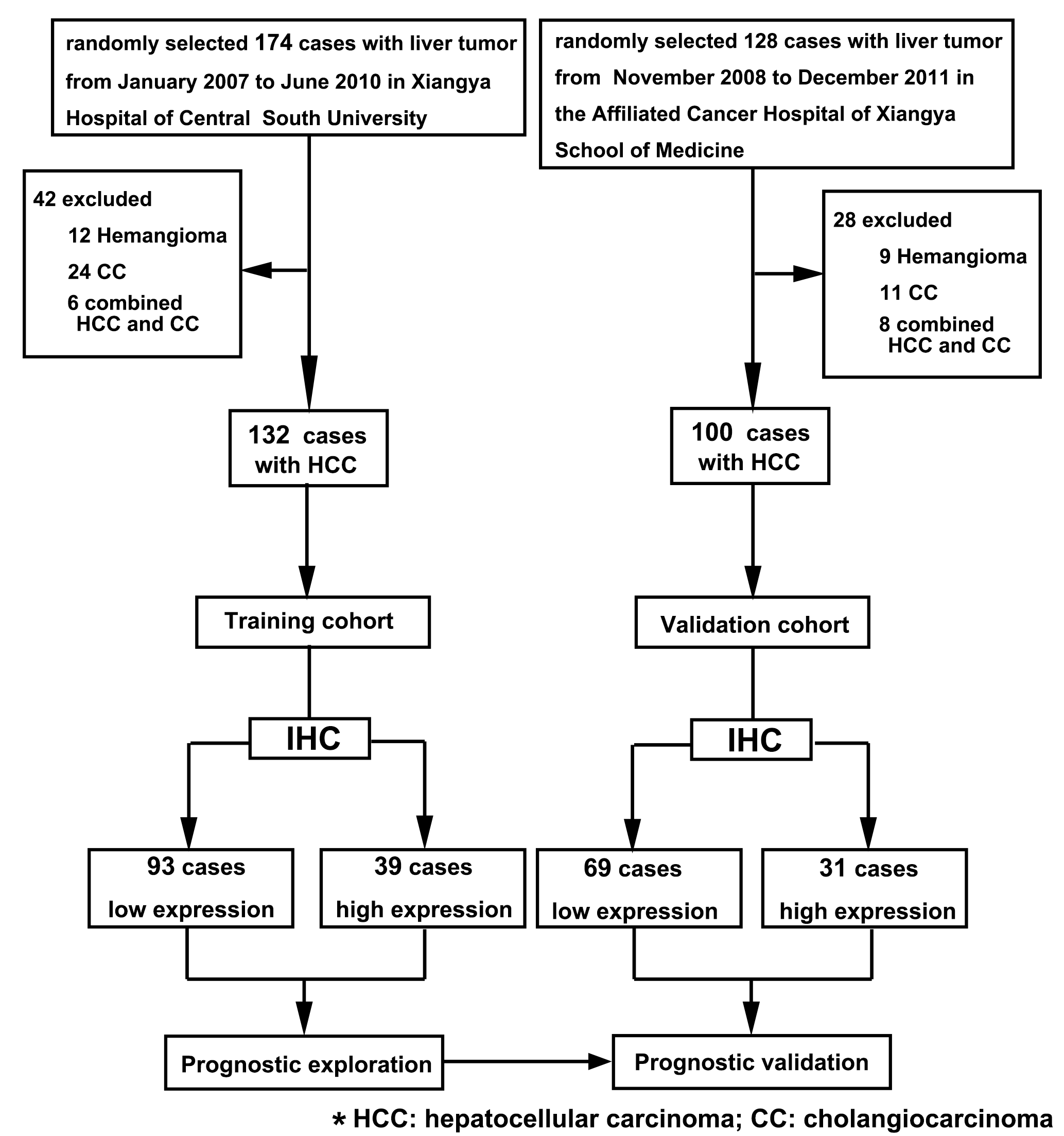

Supplement: Supplementary file 1 — Figure S1. Flow chart showing the details for selecting HCC samples in this study. (TIFF 1194 kb) [file 13046_2018_720_MOESM1_ESM.tif]

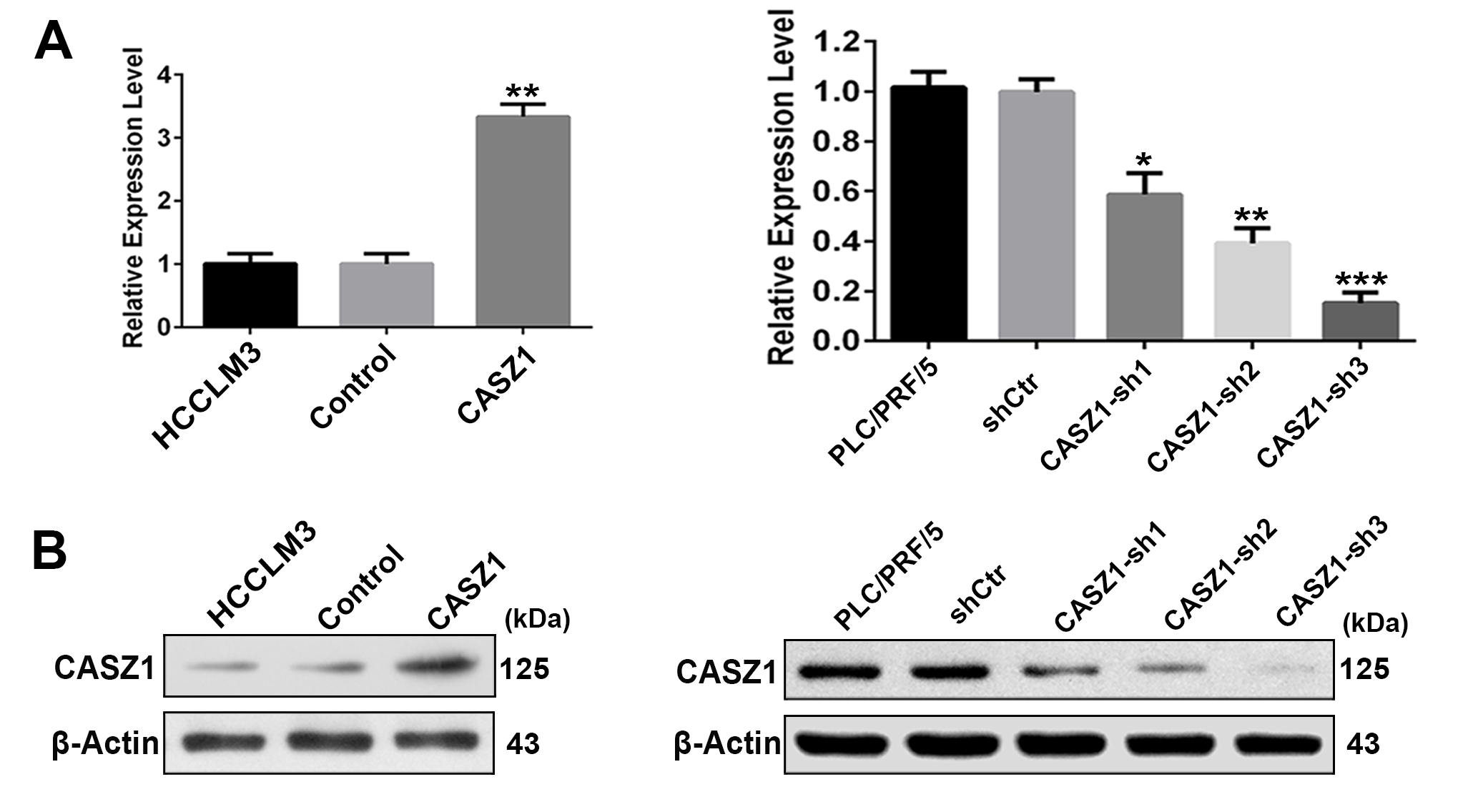

Supplement: Supplementary file 3 — Figure S2. The efficacy of CASZ1 ectopic expression or silence was determined in HCC cells. A-B. qRT-PCR (A) and western blot (B) confirmed CASZ1 mRNA and protein levels in HCCLM3CASZ1, PLC/PRF/5shCASZ1 and their respective control cells. (TIFF 8263 kb) [file 13046_2018_720_MOESM3_ESM.tif]

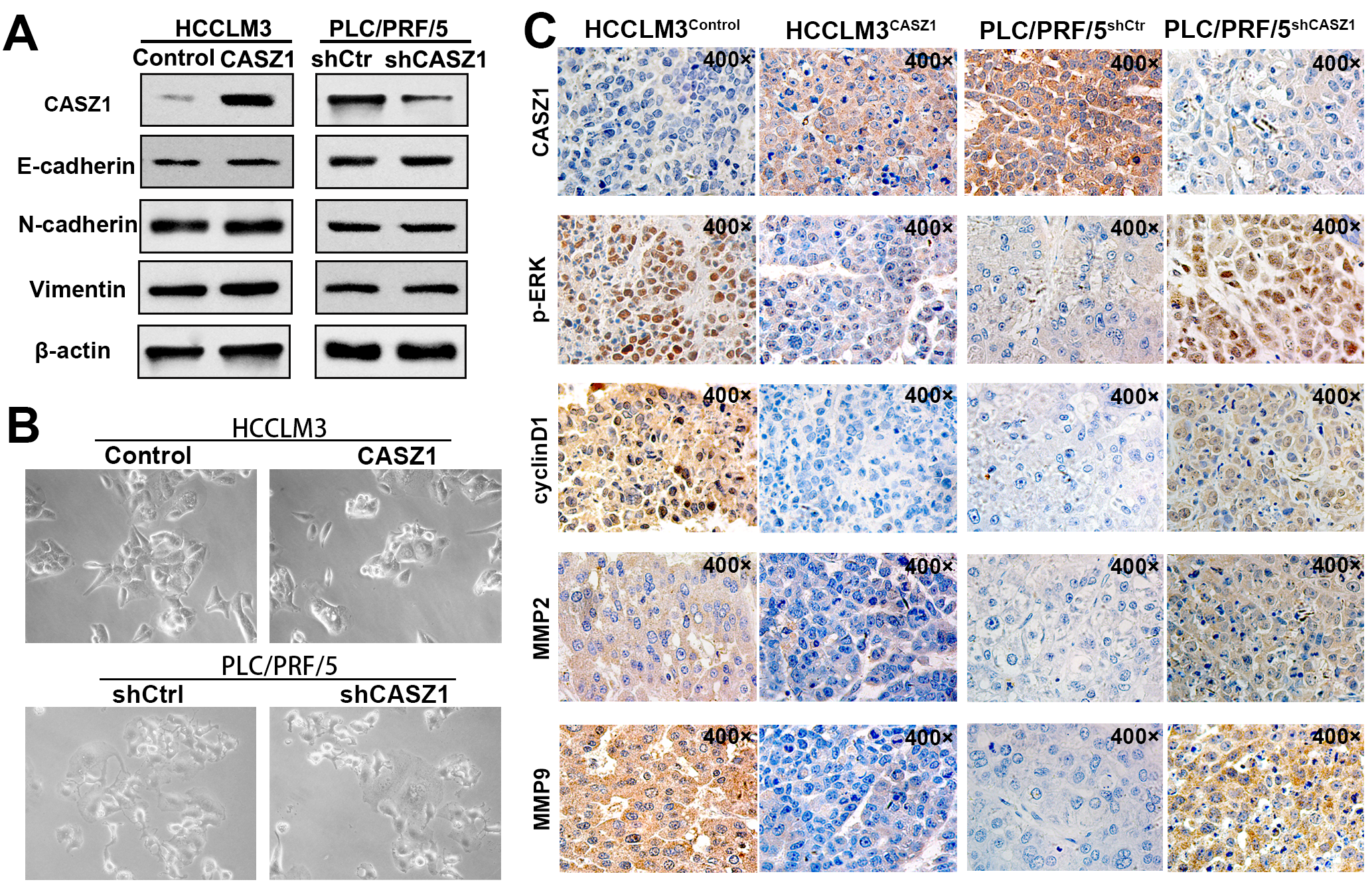

Supplement: Supplementary file 4 — Figure S3. CASZ1 inhibits HCC progression by inactivating the MAPK/ERK pathway. A EMT genes including E-cadherin, N-cadherin and vimentin were detected by western blot in HCCLM3CASZ1, PLC/PRF/5shCASZ1 and their control cells. B Cell morphological changes in HCCLM3CASZ1, PLC/PRF/5shCASZ1 and their control cells was examined by phase-contrast photomicrographs. C IHC staining showed that the expression of p-ERK, cyclinD1, MMP2 and MMP9 was reduced in the CASZ1-overexpressed HCCLM3 xenograft tumors, but increased in the CASZ1-silenced PLC/PRF/5 xenograft tumors (magnification, × 400). (TIFF 11458 kb) [file 13046_2018_720_MOESM4_ESM.tif]

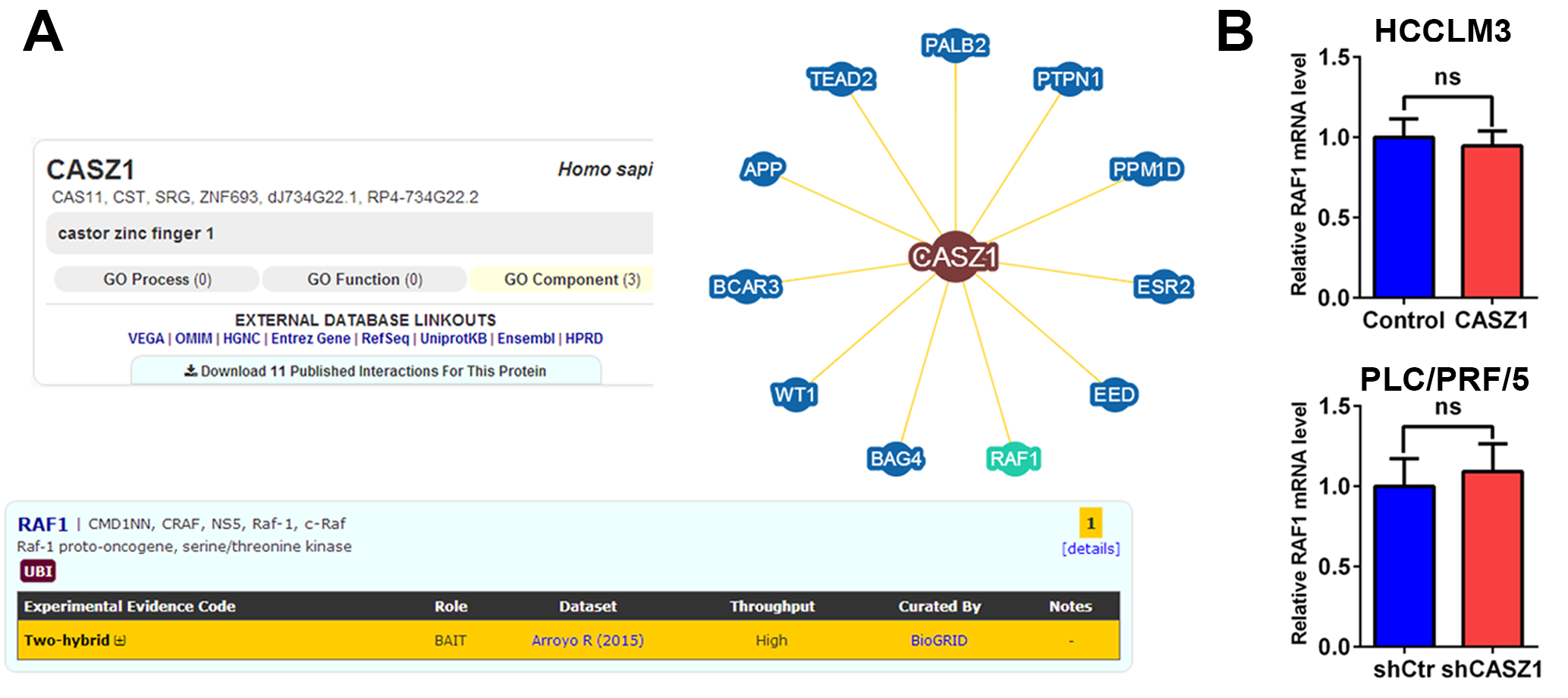

Supplement: Supplementary file 5 — Figure S4. CASZ1 may interact with RAF1 in HCC cells. A Potential CASZ1-interacting partners were analyzed using BioGRID3.4 (https://thebiogrid.org). B The expression of RAF1 mRNA was determined in CASZ1-interfered HCC cells by qRT-PCR. (TIFF 6522 kb) [file 13046_2018_720_MOESM5_ESM.tif]

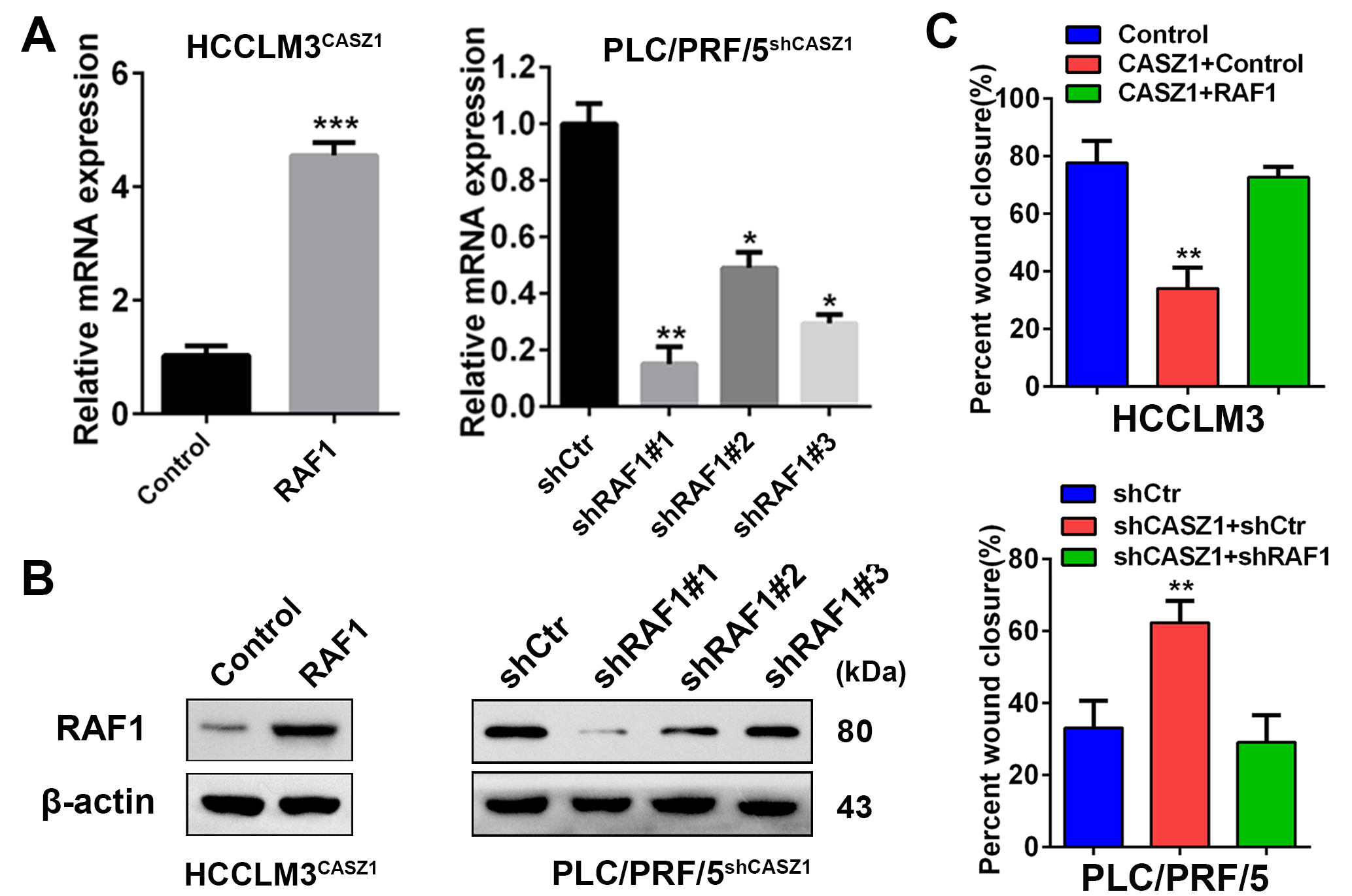

Supplement: Supplementary file 6 — Figure S5. The efficacy of RAF1 ectopic expression or silence is determined in CASZ1-interfered HCC cells. A-B. qRT-PCR (A) and western blot (B) confirmed RAF1 mRNA and protein levels in HCCLM3CASZ1 cells with RAF1 overexpression or PLC/PRF/5shCASZ1 cells with RAF1 knockdown. C. The wound closure rate of CASZ1-interfered HCC cells with RAF1 ectopic expression or knockdown. * P < 0.05, ** P < 0.01. (TIFF 2192 kb) [file 13046_2018_720_MOESM6_ESM.tif]
