# Supplementary material for: Downregulation of castor zinc finger 1 predicts poor prognosis and facilitates hepatocellular carcinoma progression via MAPK/ERK signaling
Source: J Exp Clin Cancer Res. 2018 Mar 5;37:45. doi: 10.1186/s13046-018-0720-8 (PMC5836448; doi:10.1186/s13046-018-0720-8)
Supplement: Supplementary file 2 — Table S1. Clinicopathologcal characteristics of HCC patients in training cohort and validation cohort. Table S2 Correlation between CASZ1 expression and clinicopathologic characteristics of HCC patients in training cohort and validation cohort. Table S3 Univariate and multivariate analyses of risk factors associated with overall survival and disease-free survival of HCC patients in training cohort. Table S4 Univariate and multivariate analyses of risk factors associated with overall survival and disease-free survival of HCC patients in validation cohort. (DOC 282 kb) [file 13046_2018_720_MOESM2_ESM.doc]

**Supplementary Tables**

**Supplementary Table S1. Clinicopathologcal characteristics of HCC patients in training cohort and validation cohort.**

| **Clinicopathologic**  **variables** | **Counts** | | ***P*** |
| --- | --- | --- | --- |
|  | **Training cohort** | **Validation cohort** |  |
| **Gender** |  |  | 0.740 |
| Female | 15(11.36%) | 10(10.00%) |  |
| Male | 117(88.64%) | 90(90.00%) |  |
| **Age (years)** |  |  | 0.121 |
| ≤50 | 63(47.73%) | 58(58.00%) |  |
| >50 | 69(52.27%) | 42(42.00%) |  |
| **AFP(**ng/ml**)** |  |  | 0.256 |
| <20 | 42(31.82%) | 39(39.00%) |  |
| ≥20 | 90(68.18%) | 61(61.00%) |  |
| **Hepatitis B status** |  |  | 0.453 |
| Negative | 35(26.52%) | 31(31.00%) |  |
| Positive | 97(73.48%) | 69(69.00%) |  |
| **Liver cirrhosis** |  |  | 0.670 |
| Absent | 39(29.55%) | 27(27.00%) |  |
| Present | 93(70.45%) | 73(73.00%) |  |
| **Child-Pugh classification** |  |  | 0.347 |
| A | 103(78.03%) | 83(83.00%) |  |
| B | 29(21.97%) | 17(17.00%) |  |
| **Tumor size (cm)** |  |  | 0.310 |
| ≤ 5 | 48(36.36%) | 30(30.00%) |  |
| > 5 | 84(63.64%) | 70(70.00%) |  |
| **Tumor nodule number** |  |  | 0.843 |
| Solitary | 59(44.70%) | 46(46.00%) |  |
| Multiple (≥ 2) | 73(55.30%) | 54(54.00%) |  |
| **Capsulation formation** |  |  | 0.491 |
| Presence | 38(28.79%) | 33(33.00%) |  |
| Absence | 94(71.21%) | 67(67.00%) |  |
| **Edmondson-Steiner grade** |  |  | 0.212 |
| Ⅰ&Ⅱ | 63(47.73%) | 56(56.00%) |  |
| Ⅲ&Ⅳ | 69(52.27%) | 44(44.00%) |  |
| **Microvascular invasion** |  |  | 0.252 |
| Absence | 85(64.39%) | 57(57.00%) |  |
| Presence | 47(35.61%) | 43(43.00%) |  |
| **Macrovascular invasion** |  |  | 0.961 |
| Absence | 102(77.27%) | 77(77.00%) |  |
| Presence | 30(22.73%) | 23(23.00%) |  |
| **BCLC stage** |  |  | 0.745 |
| 0&A | 28(21.21%) | 23(23.00%) |  |
| B&C | 104(78.79%) | 77(77.00%) |  |
| **TNM stage** |  |  | 0.704 |
| Ⅰ&Ⅱ | 64(48.48%) | 51(51.00%) |  |
| Ⅲ&Ⅳ | 68(51.52%) | 49(49.00%) |  |

Abbreviations: AFP, alpha-fetoprotein; HBsAg, hepatitis B surface antigen; TNM, tumor node metastasis; BCLC, Barcelona Clinic Liver Cancer.

**Supplementary Table S2. Correlation between CASZ1 expression and clinicopathologic characteristics of HCC patients in training cohort and validation cohort**.

| **Clinicopatholo-gic variables** | **Training cohort** | | | | **Validation cohort** | | | |
| --- | --- | --- | --- | --- | --- | --- | --- | --- |
|  | **n** | **CASZ1** | | ***P*** | **n** | **CASZ1 expression** | | ***P*** |
|  |  | **High** | **Low** |  |  | **High** | **Low** |  |
| **Gender** |  |  |  | 0.123 |  |  |  | 0.943 |
| Female | 15 | 7 | 8 |  | 10 | 3 | 7 |  |
| Male | 117 | 32 | 85 |  | 90 | 28 | 62 |  |
| **Age (years)** |  |  |  | 0.596 |  |  |  | 0.192 |
| ≤50 | 63 | 20 | 43 |  | 58 | 15 | 43 |  |
| >50 | 69 | 19 | 50 |  | 42 | 16 | 26 |  |
| **AFP(**ng/ml**)** |  |  |  | 0.289 |  |  |  | 0.197 |
| <20 | 42 | 15 | 27 |  | 39 | 15 | 24 |  |
| ≥20 | 90 | 24 | 66 |  | 61 | 16 | 45 |  |
| **Hepatitis B status** |  |  |  | 0.250 |  |  |  | 0.091 |
| Negative | 35 | 13 | 22 |  | 31 | 6 | 25 |  |
| Positive | 97 | 26 | 71 |  | 69 | 25 | 44 |  |
| **Liver cirrhosis** |  |  |  | 0.146 |  |  |  | 0.427 |
| Absent | 39 | 15 | 24 |  | 27 | 10 | 17 |  |
| Present | 93 | 24 | 69 |  | 73 | 21 | 52 |  |
| **Child-Pugh classification** |  |  |  | 0.793 |  |  |  | 0.674 |
| A | 103 | 31 | 72 |  | 83 | 25 | 58 |  |
| B | 29 | 8 | 21 |  | 17 | 6 | 11 |  |
| **Tumor size(cm)** |  |  |  | **0.021** |  |  |  | **<0.001** |
| ≤ 5 | 48 | 20 | 28 |  | 30 | 17 | 13 |  |
| > 5 | 84 | 19 | 65 |  | 70 | 14 | 56 |  |
| **Tumor nodule number** |  |  |  | **0.033** |  |  |  | **0.013** |
| Solitary | 59 | 23 | 36 |  | 46 | 20 | 26 |  |
| Multiple (≥ 2) | 73 | 16 | 57 |  | 54 | 11 | 43 |  |
| **Capsulation formation** |  |  |  | **<0.001** |  |  |  | **0.002** |
| Presence | 38 | 22 | 16 |  | 33 | 17 | 16 |  |
| Absence | 94 | 17 | 77 |  | 67 | 14 | 53 |  |
| **Edmondson-Steiner grade** |  |  |  | **0.005** |  |  |  | **0.043** |
| Ⅰ&Ⅱ | 63 | 26 | 37 |  | 56 | 22 | 34 |  |
| Ⅲ&Ⅳ | 69 | 13 | 56 |  | 44 | 9 | 35 |  |
| **Microvascular invasion** |  |  |  | 0.250 |  |  |  | 0.146 |
| Absence | 85 | 28 | 57 |  | 57 | 21 | 36 |  |
| Presence | 47 | 11 | 36 |  | 43 | 10 | 33 |  |
| **Macrovascular invasion** |  |  |  | 0.694 |  |  |  | 0.140 |
| Absence | 102 | 31 | 71 |  | 77 | 21 | 56 |  |
| Presence | 30 | 8 | 22 |  | 23 | 10 | 13 |  |
| **BCLC stage** |  |  |  | **0.027** |  |  |  | **0.012** |
| 0&A | 28 | 13 | 15 |  | 23 | 12 | 11 |  |
| B&C | 104 | 26 | 78 |  | 77 | 19 | 58 |  |
| **TNM stage** |  |  |  | **0.020** |  |  |  | **0.025** |
| Ⅰ&Ⅱ | 64 | 25 | 39 |  | 51 | 21 | 30 |  |
| Ⅲ&Ⅳ | 68 | 14 | 54 |  | 49 | 10 | 39 |  |

**Supplementary Table S3. Univariate and multivariate analyses of risk factors associated with overall survival and disease-free survival of** HCC patients in training cohort.

| **Clinicopatholo-gic variables** | **OS** | | | **DFS** | | |
| --- | --- | --- | --- | --- | --- | --- |
|  | **Univariate Analysis** | **Multivariate Analysis** | | **Univariate Analysis** | **Multivariate Analysis** | |
|  | ***P*** | **HR(95% CI)** | ***P*** | ***P*** | **HR(95% CI)** | ***P*** |
| **Gender** | 0.640 |  | NA | 0.913 |  | NA |
| Female |  |  |  |  |  |  |
| Male |  |  |  |  |  |  |
| **Age (years)** | 0.520 |  | NA | 0.739 |  | NA |
| ≤50 |  |  |  |  |  |  |
| >50 |  |  |  |  |  |  |
| **AFP(**ng/ml**)** | 0.344 |  | NA | 0.391 |  | NA |
| <20 |  |  |  |  |  |  |
| ≥20 |  |  |  |  |  |  |
| **Hepatitis B status** | 0.982 |  | NA | 0.273 |  | NA |
| Negative |  |  |  |  |  |  |
| Positive |  |  |  |  |  |  |
| **Liver cirrhosis** | **0.004** | **1.958(1.126-3.405)** | **0.017** | **0.002** | **1.973(1.196-3.255)** | **0.008** |
| Absent |  |  |  |  |  |  |
| Present |  |  |  |  |  |  |
| **Child-Pugh classification** | 0.496 |  | NA | 0.759 |  | NA |
| A |  |  |  |  |  |  |
| B |  |  |  |  |  |  |
| **Tumor size (cm)** | 0.058 |  | NA | 0.017 |  | **NS** |
| ≤ 5 |  |  |  |  |  |  |
| > 5 |  |  |  |  |  |  |
| **Tumor nodule number** | **<0.001** | **1.636(1.005-2.662)** | **0.048** | **0.036** |  | **NS** |
| Solitary |  |  |  |  |  |  |
| Multiple (≥ 2) |  |  |  |  |  |  |
| **Capsulation formation** | **0.004** |  | **NS** | **0.004** |  | **NS** |
| Presence |  |  |  |  |  |  |
| Absence |  |  |  |  |  |  |
| **Edmondson- Steiner grade** | **0.003** |  | **NS** | **0.013** |  | **NS** |
| Ⅰ&Ⅱ |  |  |  |  |  |  |
| Ⅲ&Ⅳ |  |  |  |  |  |  |
| **Microvascular invasion** | **0.001** | **1.694(1.087-2.640)** | **0.020** | **0.001** | **1.606(1.020-2.528)** | **0.041** |
| Absence |  |  |  |  |  |  |
| Presence |  |  |  |  |  |  |
| **Macrovascular invasion** | **<0.001** | **1.942(1.150-3.281)** | **0.013** | **<0.001** | **2.339(1.426-3.838)** | **0.001** |
| Absence |  |  |  |  |  |  |
| Presence |  |  |  |  |  |  |
| **BCLC stage** | **0.003** |  | **NS** | **0.002** |  | **NS** |
| 0&A |  |  |  |  |  |  |
| B&C |  |  |  |  |  |  |
| **TNM stage** | **<0.001** |  | **NS** | **<0.001** |  | **NS** |
| Ⅰ&Ⅱ |  |  |  |  |  |  |
| Ⅲ&Ⅳ |  |  |  |  |  |  |
| **CASZ1 expression** | **<0.001** | **1.972(1.154-3.369)** | **0.013** | **<0.001** | **2.259(1.365-3.738)** | **0.002** |
| Low |  |  |  |  |  |  |
| High |  |  |  |  |  |  |

Abbreviations: HR, hazard risk ratio; CI, confidence interval; NA, not applicable; NS, not significant.

**Supplementary Table S4. Univariate and multivariate analyses of risk factors associated with overall survival and disease-free survival of HCC patients in validation** cohort.

| **Clinicopatholo-gic variables** | **OS** | | | **DFS** | | |
| --- | --- | --- | --- | --- | --- | --- |
|  | **Univariate Analysis** | **Multivariate Analysis** | | **Univariate Analysis** | **Multivariate Analysis** | |
|  | ***P*** | **HR(95% CI)** | ***P*** | ***P*** | **HR(95% CI)** | ***P*** |
| **Gender** | 0.529 |  | NA | 0.914 |  | NA |
| Female |  |  |  |  |  |  |
| Male |  |  |  |  |  |  |
| **Age (years)** | 0.827 |  | NA | 0.365 |  | NA |
| ≤50 |  |  |  |  |  |  |
| >50 |  |  |  |  |  |  |
| **AFP(**ng/ml**)** | 0.562 |  | NA | 0.701 |  | NA |
| <20 |  |  |  |  |  |  |
| ≥20 |  |  |  |  |  |  |
| **Hepatitis B status** | 0.901 |  | NA | 0.751 |  | NA |
| Negative |  |  |  |  |  |  |
| Positive |  |  |  |  |  |  |
| **Liver cirrhosis** | **0.009** | **2.325(1.092-4.953)** | **0.035** | **0.029** | **2.163(1.076-4.349)** | **0.030** |
| Absent |  |  |  |  |  |  |
| Present |  |  |  |  |  |  |
| **Child-Pugh classification** | 0.545 |  | NA | 0.881 |  | NA |
| A |  |  |  |  |  |  |
| B |  |  |  |  |  |  |
| **Tumor size (cm)** | **0.012** |  | **NS** | **0.014** |  | **NS** |
| ≤ 5 |  |  |  |  |  |  |
| > 5 |  |  |  |  |  |  |
| **Tumor nodule number** | **0.002** |  | **NS** | **0.002** |  | **NS** |
| Solitary |  |  |  |  |  |  |
| Multiple (≥ 2) |  |  |  |  |  |  |
| **Capsulation formation** | **0.003** |  | **NS** | **0.007** |  | **NS** |
| Presence |  |  |  |  |  |  |
| Absence |  |  |  |  |  |  |
| **Edmondson- Steiner grade** | **0.040** |  | **NS** | **0.004** |  | **NS** |
| Ⅰ&Ⅱ |  |  |  |  |  |  |
| Ⅲ&Ⅳ |  |  |  |  |  |  |
| **Microvascular invasion** | **0.001** | **1.818(1.022-3.236)** | **0.042** | **0.003** | **1.827(1.049-3.182)** | **0.033** |
| Absence |  |  |  |  |  |  |
| Presence |  |  |  |  |  |  |
| **Macrovascular invasion** | **<0.001** | **2.245(1.235-4.080)** | **0.008** | **0.001** | **2.390(1.317-4.339)** | **0.004** |
| Absence |  |  |  |  |  |  |
| Presence |  |  |  |  |  |  |
| **BCLC stage** | **0.011** |  | **NS** | **0.122** |  | **NA** |
| 0&A |  |  |  |  |  |  |
| B&C |  |  |  |  |  |  |
| **TNM stage** | **0.003** |  | **NS** | **0.030** |  | **NS** |
| Ⅰ&Ⅱ |  |  |  |  |  |  |
| Ⅲ&Ⅳ |  |  |  |  |  |  |
| **CASZ1 expression** | **0.002** | **2.965(1.426-6.165)** | **0.004** | **0.001** | **3.093(1.535-6.233)** | **0.002** |
| Low |  |  |  |  |  |  |
| High |  |  |  |  |  |  |
